# Use of Multivariate Analysis to Unravel the Differences between Two Chamomile Varieties and Their Anticancer and Antioxidant Activities

**DOI:** 10.3390/plants12122297

**Published:** 2023-06-12

**Authors:** Dana Atoum, Ignacio Fernandez-Pastor, Louise Young, RuAngelie Edrada-Ebel

**Affiliations:** 1 Strathclyde Institute of Pharmacy and Biomedical Sciences, University of Strathclyde, Glasgow G4 0RE, UK; dana.atoum@hu.edu.jo (D.A.); ignaciofernandezpastor@gmail.com (I.F.-P.); louise.c.young@strath.ac.uk (L.Y.); 2 Department of Pharmaceutical Chemistry, Faculty of Pharmaceutical Sciences, The Hashemite University, Zarqa 13133, Jordan; 3 Fundación MEDINA, Centro de Excelencia en Investigación de Medicamentos Innovadores de Andalucía, Parque Tecnológico de Ciencias de la Salud, Avda. del Conocimiento 34, 18016 Granada, Spain

**Keywords:** anticancer, antioxidant, biological activities, NMR, LC-MS, metabolomics, multivariate analysis, chemical profiling, chamomile

## Abstract

Background: Plants from the Asteraceae family were commonly used to treat various diseases. The metabolomic profile of this family consisted of bioactive flavonoids and other phenolics. Chamomile is a member of the Asteraceae family. Jordanian and European chamomile are two varieties of *Matricaria chamomilla* (German chamomile), which were grown under different environmental conditions, were studied. Many examples of plant varieties with significant distinction in the secondary metabolite they afford have been described in the literature. Multivariate statistical analysis was employed to measure the depth of this variation in two chamomile varieties. Methods: From both types, crude extracts were prepared using solvents of different polarities and tested for their biological activity. The semipolar fraction of the European variety showed anticancer and antioxidant activity. Meanwhile, the semipolar fraction of the Jordanian type exhibited only antioxidant activity. Both extracts were fractionated, and then the biological activity was again assayed. Results: European and Jordanian chamomile fractions produced dicaffeoylquinic acid isomers exhibiting antioxidant capability. Additionally, *Z*-glucoferulic acid was produced from the European chamomile, demonstrating antioxidant activity. The European samples afforded two major compounds, chrysosplenetin and apigenin, that displayed anticancer activity. Conclusions: Different environmental conditions between Jordanian and European chamomile affected the type of isolated compounds. Structure elucidation was performed with HPLC-MS coupled with dereplication techniques and 2D NMR experiments.

## 1. Introduction

Environmental conditions closely affect the biosynthesis of the primary and secondary metabolites in specimens of the same plant [1,2]. In many instances, plants’ physiological adaptations are under the influence of the ecosystem that surrounds them [3]. Secondary metabolites play different roles in the plant interactions, such as competition, symbiosis, metal transport, differentiation, etc. [4]. Some volatile terpenes are involved in plant communication and defensive system [5,6]. Plant polyphenols have multiple functions as UV-screening pigments or counter oxidative stress agents [7]. Herbal infusions have historically been the main form of drugs in traditional medicine. Chamomile is a native flowering plant from Europe and Western Asia [8]. Flower brews have been used to treat many diseases for thousands of years [9]. This plant has been escribed for its many beneficial health effects, such as anti-inflammatory, mild sedative, antioxidant, anticancer, neuro-protective, anti-allergic and anti-microbial [10,11,12,13,14,15]. 

Structural elucidation reports of chamomile flower constituents have allowed us to identify approximately 120 secondary metabolites, including 28 types of terpenoids and 36 polyphenolic compounds [16]. Most important constituents attributed to therapeutic properties have been correlated to their secondary metabolites, such as phenolic acids, flavonoids, coumarins, sesquiterpenes, and polyacetylenes [17,18,19]. Most known chamomile polyphenols have been derived from the biologically active flavonoid apigenin, which included therapeutic congeners that were methylated, acetylated, and glycosylated [20,21,22].

The advances in the sensibility, selectivity, and resolution of HPLC-MS instruments combined with the structural information obtained from NMR experiments allowed metabolomic profiling of complex samples [23,24,25]. The HPLC-MS data was processed by the open-source software Mzmine 2. Such toolboxes would accomplish peak picking, peak list deisotoping, alignment, gap filling, and normalization of the mass spectral dataset [26]. Dereplication was then achieved to screen known natural products from a comprehensive database such as the Dictionary of Natural Products (DNP).

Multivariable data analysis (MVDA) is the optimum tool to visualise metabolite distributions from different sample varieties. Principal component analysis (PCA) was used to detect characteristic compounds in an unlabelled analytical dataset. The main advantages of this statistical model include reducing dataset dimensions and increasing interpretability while minimizing information loss [27]. PCA scores plots are a conceptually simple 2D projection of datasets to express sample-group similarities. They identify sample outliers that could be interesting or be sorted out to correct errors of unknown variation in data collection. On the other hand, statistical models using orthogonal partial least square data analysis (OPLS-DA) assign datasets in two classes to cluster the influence of their metabolic fingerprints while defining the differences between them [28]. This current study used the OPLS-DA loadings S-plot to predict the bioactive metabolites from the antioxidant and anticancer active fractions between the two chamomile varieties. Biological activity testing of raw extracts and crude fractions showed in which polarity the active compounds were present. The fractionation of those extracts combined with metabolomics analysis accelerated the search for promising compounds.

This study explored the variations in biologically active compounds between two varieties of the same species grown under different environmental conditions. We aimed to (1) employ HPLC-MS and NMR to distinguish the metabolomics fingerprint in both varieties of chamomile; (2) distinguish patterns in the types of secondary metabolites present in both samples in terms of their bioactivities; and (3) identify the bioactive compounds in the Jordanian and European variety.

## 2. Results

### 2.1. Crude Extracts Results

**Figure 1:**
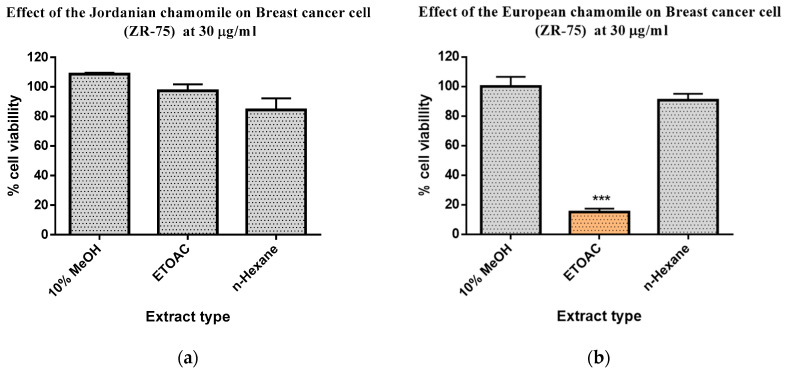
AlamarBlue^®^ essay on breast cancer cell line ZR-75 of 30 µg/mL crude fractions obtained by liquid-liquid partitioning of total crude extracts of (**a**) Jordanian and (**b**) European chamomile. *n* = 3, ***: *p* < 0.001 means statistically significant. *p*-value determined by ANOVA test.

The same extraction procedures were employed for both Jordanian and European chamomile varieties. Flower parts were used for both varieties. Liquid-liquid partitioning was performed on dried total crude acetone-methanolic extracts to obtain the aqueous MeOH, n-Hexane, and EtOAc extracts. The obtained weights of the dried n-Hexane, aqueous MeOH, and EtOAc extracts from the Jordanian chamomile were 0.19 g (1.9%), 9.6 g (96%), and 0.18 (0.18%), respectively. Meanwhile, the European chamomile afforded 0.3 g (0.3%) of the n-Hexane extract, the aqueous MeOH extract was 9.9 g (99%), and the EtOAc extract was 0.34 g (0.34%). From both varieties, the highest extract yields were attained with aqueous MeOH. The solvent-partitioned extracts were then bio-assayed for antioxidant and anticancer screening against a breast cancer cell line ZR-75 (Figure 1). 

AlamarBlue^®^ (Biosource International, Carmillo, CA, USA) was used to determine the viability of the cancer cells in the presence of the crude plant solvent-partitioned fractions. Only the EtOAc extract of the European variety significantly exhibited activity against the breast cancer ZR-75 cell line at 89% (*p* < 0.0001), that is, cell viability of 11% (Figure 1). On the other hand, antioxidant activity was observed for the EtOAc extracts of both European and Jordanian varieties, as shown in Table 1. Data were then expressed as mean ± SEM and were plotted using GraphPad Prism Software version 6.0 for Windows (GraphPad Software, La Jolla, CA, USA, www.graphpad.com, accessed on 1 May 2020). The antioxidant activity was measured using a DPPH assay. Ascorbic acid and quercetin were used as positive controls. EC_50_ (half maximal effective concentration) values were determined for both bioactive extracts, equal to 0.16 and 0.2 mg/mL for the Jordanian and European chamomile EtOAc extracts, respectively. Both n-Hexane and aqueous MeOH extracts were inactive in the AlamarBlue^®^ and DPPH assays.

### 2.2. Multivariate Analysis of NMR Spectral Data

**Figure 2:**
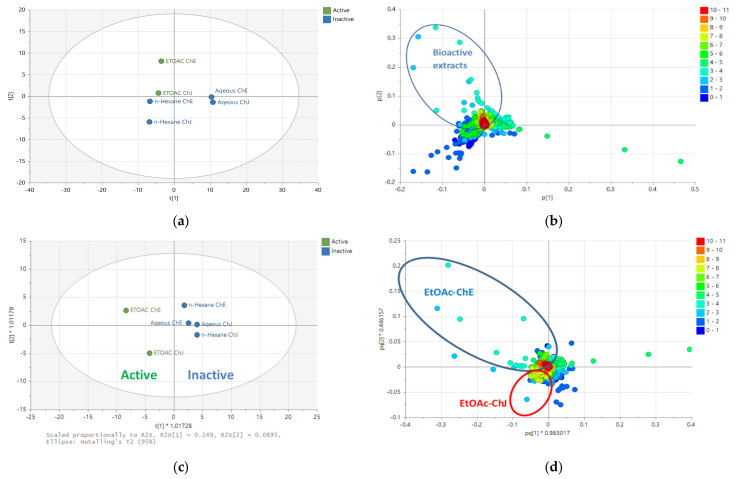
(**a**) PCA scores and (**b**) loadings plots of the NMR spectral data of *M. chamomilla*. The R^2^ and Q^2^ values were 0.98 and 0.90, respectively. Encircled features on the loadings plot indicate the discriminating chemical shifts for the biologically active extracts. (**c**) OPLS-DA scores and (**d**) loadings plots of the NMR spectral data of *M. chamomilla* pre-classified according to their bioactivities in AlamarBlue^®^ and DPPH assay results. The R^2^ and Q^2^ values were at 0.98 and 0.80, respectively. Encircled features on the loadings plot indicate the discriminating chemical shifts for the biologically active extracts. (ChJ = Jordanian; ChE = European).

The AlamarBlue^®^ and DPPH assay results were incorporated with the NMR spectral dataset for multivariate analysis. The PCA scores scatter plot naturally grouped the bioactive EtOAc extracts in the upper left quadrant, indicating the uniqueness of the European variety (EtoAc ChE). The n-Hexane extracts were positioned in the lower left quadrant. The aqueous MeOH extracts of both varieties were grouped at the right quadrants of the plot, suggesting their similar chemical profiles (Figure 2a). The loadings plot of NMR data showed that these bioactive extracts contained highly acetylated, methoxylated, and glycosylated metabolites, as implied by the ^1^H resonances at 2.00 to 4.00 ppm (encircled in blue), as shown in Figure 2b. The inactive hexane extracts were dominated by chemical shifts between 0 and 2 ppm (blue dots) for lipids, as displayed on the lower left quadrant. The R^2^ was 0.98, and Q^2^ was 0.90 after six components, which indicated a good-fitted model with good predictability. The R^2^ indicates the model’s fitness, and Q^2^ indicates the predictive ability of the model.

For the OPLS-DA scores plot (Figure 2c), the extracts were pre-classified according to their bioactivity results in the AlamarBlue^®^ and DPPH assays. The bioactive EtOAc extracts of Jordanian (ChJ) and European (ChE) varieties dispersed in the lower and upper left quadrants, respectively. This implied the difference in chemical profiles for the respective bioactive extracts. On the other hand, the inactive extracts were in the right quadrants of the scores plot. For the OPLS-DA loadings plot illustrated in Figure 2d, the EtOAc extracts for Jordanian chamomile revealed the presence of metabolites with chemical shifts resonating between 6.00 and 8.00 ppm, while a few discriminating acetylated resonances at 2.0 to 3.0 ppm (encircled in red) were also evident.

Meanwhile, the NMR spectral data of European chamomile extracts exhibited a higher density of resonances between 2.0 and 4.0 ppm, indicating the presence of acylated and hydroxylated or glycosylated active metabolites (encircled in blue). On the other hand, the n-Hexane and aqueous extracts for both chamomile varieties were distributed in the right quadrants of the OPLS-DA scores plot. The corresponding loadings plot exhibited the presence of resonances between 0.00 and 5.00 ppm for the inactive extracts, suggesting the presence of lipids and glycosylated compounds. The R^2^ was 0.98, and Q^2^ was 0.80, indicating good fitness and predictability. The variation score between groups was 24.9% but just 8.9% within groups, indicating lesser diversity within the respective groupings but a more defined difference between the active and inactive extracts.

### 2.3. Multivariate Analysis of LC-HRMS Data

**Figure 3:**
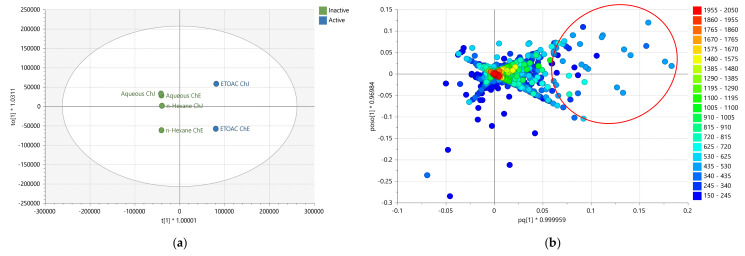
(**a**) OPLS-DA scores and (**b**) loadings plot for the mass spectral data (ion peaks at *m*/*z*) of the crude extracts obtained by liquid-liquid partitioning (ChJ = Jordanian; ChE = European). Encircled features in red indicate the discriminating features for the active EtOAc extracts, listed below in Table 2. The R^2^ and Q^2^ values were 1.00 and 0.96, respectively.

Similarly, the results from the anticancer and antioxidant assays were incorporated with the LC-MS data for multivariate analysis. The extracts were grouped according to their bioactivity. The active extracts were allocated on the right quadrants of the OPLS-DA scores plot, while the inactive extracts were on the left quadrants, as shown in Figure 3a. The biologically active EtOAc extracts from Jordanian and European chamomile were distributed in the lower and upper left quadrants, indicating different chemical profiles for the respective varieties, which correlates with the NMR spectral dataset. The inactive extracts included aqueous MeOH and n-Hexane extracts from both varieties. The OPLS-DA model gave validation scores of 1.00 and 0.96 for fitness (R^2^) and predictability (Q^2^), respectively, that afforded a difference of less than 0.3, indicating a strong model without overfitting. The variation R^2^X_o_ [1] between groups was 25.9%, while R^2^X [1] was 41.2% within groups. The large variation score within the respective groups suggested quite a diverse chemical profile between each extract in terms of their mass spectral data. From the OPLS-DA loadings plot (Figure 3b), the bioactive metabolites displayed a *m*/*z* value range between 270 and 600 Da, indicating a medium molecular mass, which supported the ^1^H NMR data providing probable evidence for the presence of phenylpropanoids and flavonoid compounds. The inactive extracts showed ion peaks for lower MWs compounds between 150 and 340 Da.

**Figure 4:**
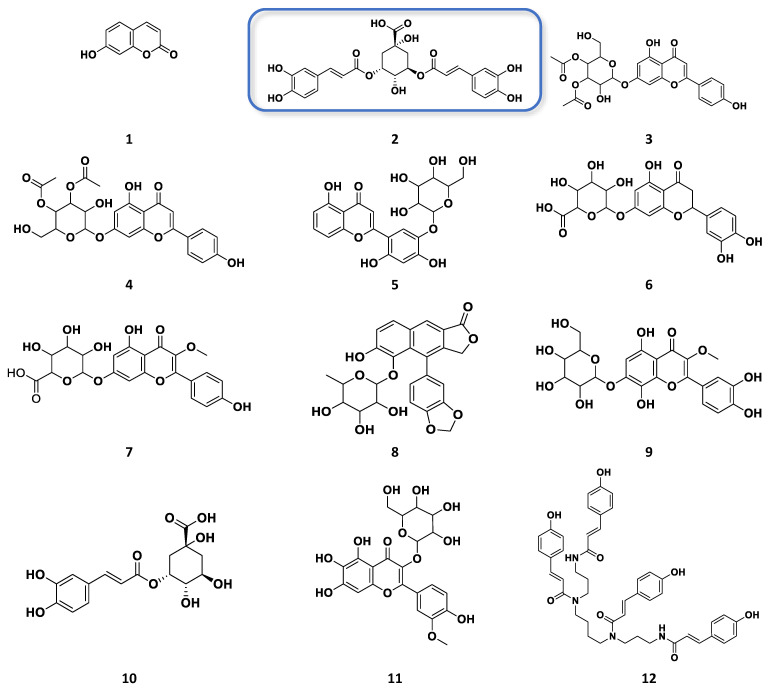
Structures of the discriminating bioactive metabolites detected from the EtOAc extracts of both Jordanian and European varieties of *M. chamomilla* as listed in Table 2. Structures in boxes were the isolated compounds in this study.

OPLS-DA loadings plot (Figure 3b) afforded the discriminating metabolites encircled in red, while those with *p*-values < 0.05 were listed in Table 2, and their structures were shown in Figure 4. These included N_3285 and N_3284 with [M − H]^−^ ion peaks found at *m*/*z* 353.0880 and 515.1196 Da, respectively, that were derivatives of caffeoyl quinic acid, which have been described for their antioxidant activity [29]. These metabolites were found in the Jordanian chamomile EtOAc extract. Furthermore, discriminating metabolites with MZmine ID P_7541, P_21550, P_845, and N_3306, with *m*/*z* values of [M + H]^+^ 449.1079, 465.1028, 465.1029, 495.1133, and [M − H]^−^ 463.0887 Da, respectively were dereplicated as flavonoid derivatives. Numerous flavonoids have been shown to have anticancer properties. However, the molecular mechanisms behind this effect have not been completely understood [30]. The latter metabolites were found in the European chamomile EtOAc extract, which supported the evidence for its anticancer activity against breast cancer cells. It was reported earlier that the antioxidant activity correlates with total phenolic content, not total flavonoids [31].

### 2.4. NMR Analysis of M. chamomilla Fractions

**Figure 5:**
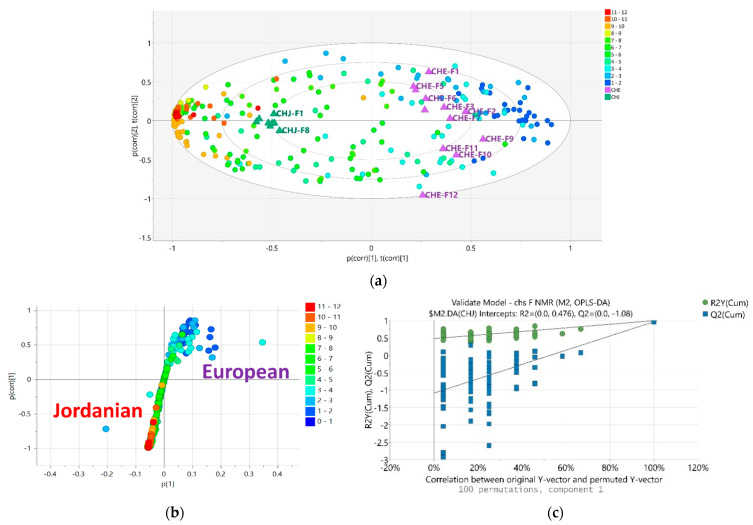
(**a**) PCA Biplot and (**b**) OPLS-DA loadings S-plot of the NMR spectral data of Jordanian and European chromatographic fractions. (**c**) A permutation test (100 permutations) of the OPLS-DA model for chromatographic fraction bioactive EtOAc extracts of Jordanian versus European varieties.

The resulting active extracts were further subjected to chromatographic isolation work. The same fractionation methodologies were employed for both the active semi-polar extracts of the two chamomile varieties. The PCA Biplot (scores or observations are in triangles and chemical shifts features in circles) of the NMR spectral data of the chromatographic fractions of the EtOAc extracts (Figure 5a) showed obvious differences between the European and Jordanian varieties. The fractions obtained from the Jordanian herb were quite homogeneous and closely clustered, which implied a strong similarity in their chemical profiles, which were highly phenolic with the presence of their aromatic hydroxyl resonances between 9 and 12 ppm [32]. Quite the opposite, the European fractions were more dispersed and diverse. While the absence of exchangeable hydroxyl shifts found in the European samples indicates the glycosylation of the hydroxyl moieties with increasing density of resonances between 3.0 and 5.0 ppm as shown on the loadings S-plot between the two varieties (Figure 5b). Additionally, the ^1^H NMR spectra of polar fractions ChE9 to ChE12 revealed significant differences from the earlier eluted non-polar fractions. Several chemical shifts from the aliphatic alkyl region (1.0 to 3.0 ppm) were absent in the most polar fractions of the European chamomile, which afforded higher density of glycosylated or methylated phenolic resonances between 3 and 7 ppm. This was in accordance with the separation of the more polar fractions observed in the PCA scores plot of their NMR spectral data, as shown in Figure 5a. In the generated model, R^2^ was 0.92, and Q^2^ was 0.58, suggesting a model with good fitting but predictability was of medium strength, which was caused by the dispersion of the fractions from the European chamomile extract.

Comparative assessment of the NMR spectral data of the fractions was done by OPLS-DA. As illustrated by an S-plot (Figure 5b), the European chamomile afforded more glycosylated or methoxylated compounds unique to the variety, implied by the discriminating peaks between 3 and 5 ppm. On the other hand, the discriminating features for the Jordanian variety were represented by a higher density of resonances between 6 and 7 ppm, which complied with polyphenolic proton chemical shifts [33]. Validation scores of the model gave an R^2^ value of 0.97 and a Q^2^ of 0.95, whereas the R^2^Y intercept was at 0.99 and the Q^2^Y intercept was at −1.04. These numbers suggested both excellent fitting and prediction. The difference between R^2^ and Q^2^ values was 0.02, indicating that the model was not overfitted. The validity of the model was further confirmed by permutation. The fact that Q^2^Y was negative, −1.08, provided more evidence of its validity (Figure 5c). Furthermore, the percentage variation R^2^X_o_ [1] between groups is 34.2%, while R^2^X [1] is 9% within groups.

### 2.5. LC-HRMS Analysis of M. chamomilla Fractions

HRMS assisted in the dereplication of the metabolites found in the fractions obtained by flash chromatography. NMR experiments on the fractions complemented the putative identification of known compounds. Furthermore, the distribution of metabolites in both varieties sorted by their MW showed some significant differences between samples.

**Figure 6:**
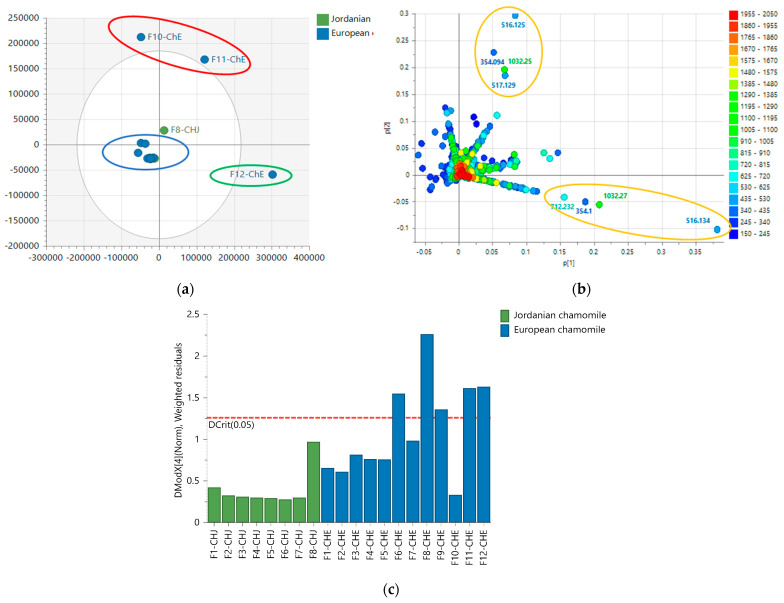
(**a**) PCA scores and (**b**) loading plots of the LC-HRMS data of *M. chamomilla* fractions for both varieties. Encircled in orange are the discriminatory features for the corresponding outlying variable fractions and listed in Table 3. (**c**) DMod X results to validate true outliers of the model. Variables above the red line are the true outliers, including Fractions ChE 6, 8, 9, 11, and 12.

In the first approach, the PCA model (Figure 6) was based on the MW distribution of the metabolites found in both chamomile varieties. The PCA scores plot of the LC-HRMS data of both varieties revealed that fractions obtained from the Jordanian herb were overlappingly clustered together, indicating a strong similarity in metabolomic profiles between all these fractions. Parallel to the indicative results obtained from the NMR spectral data, fractions from the European variety were dispersed with outlying polar fractions F10, F11, and F12. (Figure 6a). The European chamomile fractions could be classified into three groups: Fractions 1 to 9 (encircled in green), Fractions 10 and 11 (encircled in blue), and Fractions 12 (encircled in red). However, the less polar Fractions 1 to 9 overlapped with the Jordanian chamomile fractions, indicating similarity in their chemical profile.

**Figure 7:**
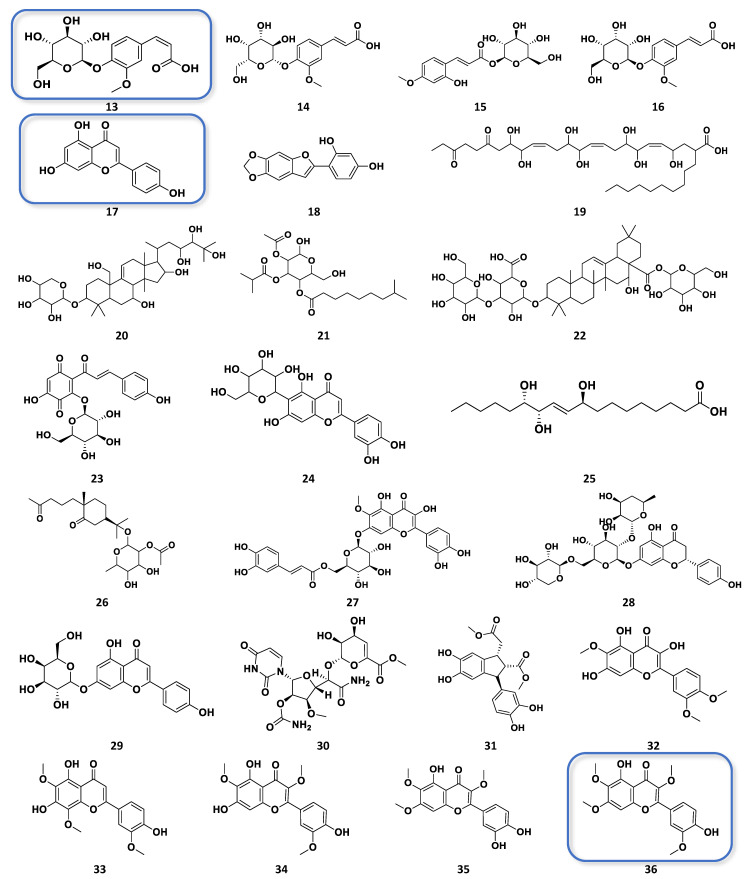
Structures of the discriminating and target bioactive metabolites against breast cancer (ZR-75) and antioxidant activity as listed in Table 3. Structures in boxes were the isolated compounds in this study as detected from the total ion chromatogram (TIC), as shown below in Figure 8.

**Figure 8:**
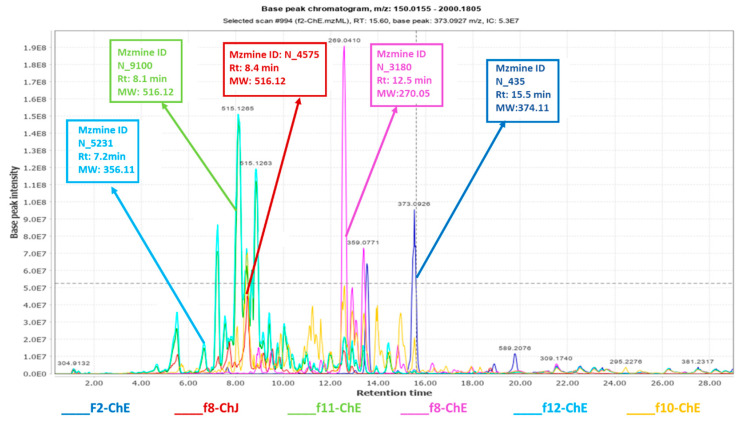
Total Ion Chromatogram (TIC) of the active fractions. The labelled ion peaks represent the semi-purified discriminating features validated by 2D NMR, highlighted in Table 3. (The inactive outlier f10_ChE is shown here compared to the outlying active fraction f11_ChE, both observed on upper quadrants of the PCA plot in Figure 6a).

Despite the high score of 0.961 for the goodness of fit (R^2^), the PCA model (Figure 6) has a low predictability score (Q^2^) of 0.356 after nine components. Polar fractions F10, F11, and F12 were presented as outliers (Figure 6a), indicating a strong likelihood that they had a more distinct profile when compared to the other fractions in the group. The loadings plot in Figure 6b demonstrated the discriminatory features for these outlying fractions. F10-ChE and F11-ChE were discriminated by metabolites with MWs of 354.094 (N_3484), 516.125 (N_9100 and N_4575), 517.129 (N_11743) and 1032.25 (N_5222) Da. Whereas F12-ChE was differentiated by metabolites with MWs of 354.099 (N_2089), 516.134 (N_14912), 712.232 (N_14913) and 1032.27 (N_14914) Da. These discriminatory metabolites were annotated as listed in Table 3, and their structures are shown in Figure 7. All these variable features were later designated as the target antioxidant metabolites for the bioactive fractions F11-ChE and F12-ChE from the European variety, as presented under Section 2.6. However, F10-ChE was found to be inactive. As illustrated in Figure 8, despite the occurrence of the target bioactive metabolites in F10-ChE, with N_4575, eluting at 8.4 min as one of its major components amongst the fraction’s unique variable features, all other defined discriminating metabolites have a very low relative abundance that would be below their potency threshold [34].

Furthermore, F10-ChE, F11-ChE, and F12-ChE were evaluated to determine whether they were real outliers using DModX. Fractions 11 and 12 were identified as true outliers, indicating a high possibility of having a more distinct profile when compared to the other fractions. The DModX plot (Figure 6c) shows that variables above the red line are true outliers, including Fractions 6, 8, and 9. However, F10 was not classified as a “true” outlier.

**Figure 9:**
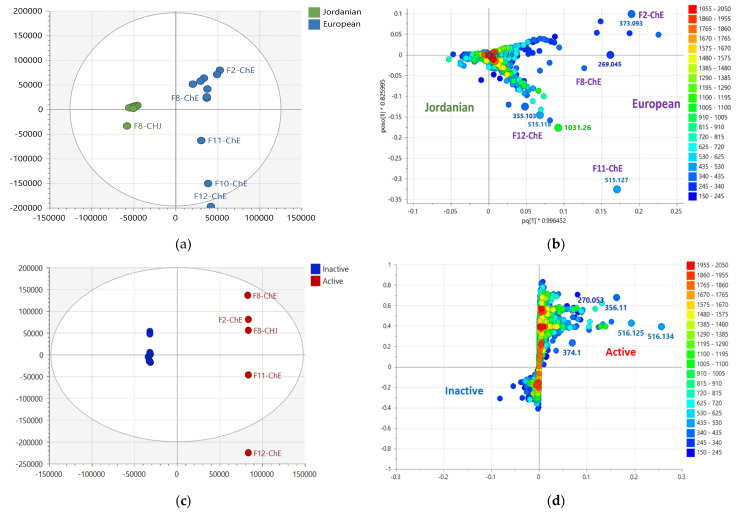
(**a**) OPLS-DA scores and (**b**) loadings plots of the *m*/*z* ion peaks grouped between Jordanian and European chamomile fractions. R^2^ and Q^2^ scores were 0.976 and 0.88, respectively. (**c**) OPLS-DA scores plot and (**d**) loadings S-plot of the mass spectral data (in MW) grouped between active and inactive fractions. R^2^ and Q^2^ values are equal to 1.0 and 0.41, respectively. The discriminatory features are presented in Table 3.

For a more accurate observation of the chemical variations between both varieties, an OPLS-DA showed further evidence for the difference between the metabolomic profiles of the two varieties (Figure 9). The presence of metabolites with a wider range of MWs from 150 to 1600 Da was found in the European variant. Whereas the Jordanian variant afforded a narrower MW range of metabolites between 600 and 800 Da (Figure 9a).

The loadings plot was generated from the OPLS-DA scores plot model (Figure 9b). The discriminating features for the European variety with ion peaks [M − H]^−^ at *m*/*z* 355.103, 373.093, 269.045, 515.127, 515.118 and 1031.260 Da were associated with the active fractions corresponding to F2-ChE, F8-ChE, F11-ChE and F12-ChE, respectively. Interestingly, ion peaks [M − H]^−^ at *m*/*z* 515.127 and 515.118 Da were already pinpointed as the discriminating features for the active EtOAc extracts, and 1031.260 is a complex of *m*/*z* 515.127 Da. Determination of the bioactivity of the fractions is presented under Section 2.6. The generated model has an R^2^ of 0.97 and a Q^2^ of 0.88. These values suggested that the fitting and prediction were valid. Additionally, the difference between Q^2^ and R^2^ was 0.072, less than 0.3, suggesting no overfitting occurred. The variation between the two groups R^2^X_o_ [1] was 21.8%. While the variation within group R^2^X [1] was at 8.9%, which indicated that the fractions within the two classes were very similar in their chemical profile. While on the other hand, there is a defined difference between the Jordanian and European variants since the variance score between groups is significantly bigger than the variance score within groups. 

### 2.6. Biological Assay Results of the Bioactive Fractions

The resulting 12 major fractions from the European chamomile and eight major fractions from the Jordanian variety were tested using the DPPH assay to determine antioxidant activity. F11 and F12 from the European variety, while only F8 from the Jordanian variety showed antioxidant activity (Figure 9c). The inactive fractions of both varieties clustered together, that suggested their similarity in chemical profiles. The EC50 values of each bioactive fraction were calculated, as shown in Table 4. Fraction 8 of the Jordanian variety was the most potent antioxidant among all the fractions, which afforded an EC50 value of 0.076 mg/mL.

On the other hand, Fractions 11 and 12 showed antioxidant activity with EC50 values of 0.311 and 0.165 mg/mL, respectively. Quercetin and ascorbic acid were positive controls that exhibited EC50s of 0.146 and 0.034 mg/mL, respectively. In the literature, ascorbic acid and quercetin were used as a positive control because they have potent antioxidant activity with EC50 values of 0.11 and 0.012 mg/mL, respectively [35,36]. The European variety’s segregated fractions F11 and F12, as observed on the lower right quadrant of the OPLS-DA scores plot (Figure 9c), gave a relatively good antioxidant activity comparable to ascorbic acid. From the OPLS-DA loadings S-plot (Figure 9d), F11 and F12 pointed out the bioactive target metabolites 7″-*Z*-3,5-di-*O*-caffeoylquinic acid (N_9100) and *Z*-glucoferulic acid (N_5225) with significant *p*-values of 0.015 and 0.002, respectively (Table 3). On the other hand, N_4575 eluting at 8.47 min (Figure 8) from F8 of the Jordanian variety is a structural isomer of N_9100 and was relatively parallel to quercetin’s antioxidative strength despite a higher *p*-value of 0.077.

Moreover, fractions of both varieties were tested against both breast cancer (ZR-75). The cell viability was measured at a sample concentration of 30 µg/mL. Only fractions F2-ChE and F8-ChE of the European variety exhibited anticancer activity against the breast cancer cell line ZR-75, as observed on the upper right quadrant of the OPLS-DA scores plot (Figure 9c). F2 and F8 showed 17% and 21% cell viability, respectively.

**Figure 10:**
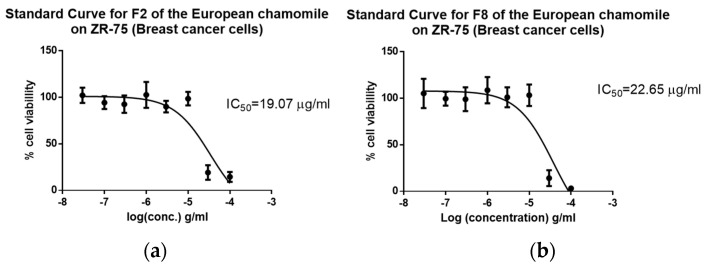
Standard curves to determine the IC_50_ values for the bioactive fractions (**a**) F2 and (**b**) F8 from the European chamomile tested against the breast cell line ZR-75.

**Figure 11:**
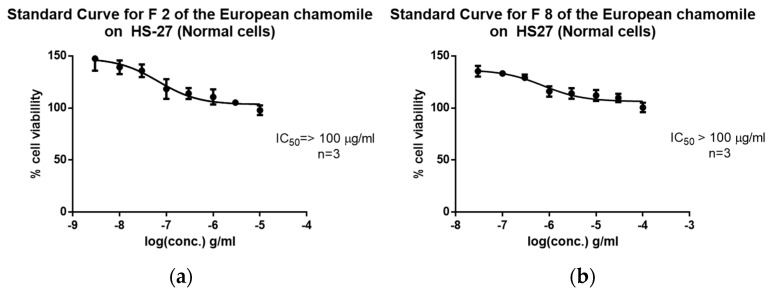
Standard curves to determine the IC50 values for the bioactive fractions (**a**) F2 and (**b**) F8 from the European chamomile tested against the normal cell line HS-27.

Furthermore, using eight concentrations, the IC_50_ values against the breast cancer cell line ZR-75 were determined for the bioactive fractions F2-ChE and F8-ChE. F2-ChE exhibited an IC_50_ of 21.07 µg/mL, while F8-ChE showed an IC_50_ of 22.65 µg/mL (Figure 10). The isolated bioactive fractions were also assayed for their toxicity against human foreskin fibroblast cell line HS-27. The IC_50_ value for both F2-ChE and F2-ChE against HS-27 were not obtainable (Figure 11). The IC50 values of both samples were in the ppm range, µg/mL, which measures concentration. The selectivity index (SI) was calculated by dividing the IC50 of the tested normal cell over the IC50 of the tested cancer cells. If the SI value was greater than 3 or if the IC50 value of a compound against the normal HS-27 cell line could not be determined in the concentration range used, then this suggested that the tested compounds were considered selective [37].

The selectivity index for F2-ChE and F8-ChE were ≥4.74 and 4.41, respectively, which indicated both fractions were selective. F2-ChE and F8-ChE were found selective against the tested breast cancer cell line and were not toxic against the normal cell line HS-27 (Table 5). As determined from the OPLS-DA loadings plot (Figure 9D), F2-ChE and F8-ChE yielded target bioactive metabolites chrysoplenetin (N_435) and apigenin (N_3180) at *p-*values of 0.3460 and 0.0137, respectively (Table 3). Apigenin displayed its significance (*p* < 0.05) as a potential anti-oncogenic compound against the breast cancer cell line ZR-75.

Distinctively, the target bioactive metabolites were remarkably detected on the negative mode of ionization, indicating the phenolic structure or acidic nature of the compounds. N_9100, N_4575 and N_3484 were putatively dereplicated in Table 3 as either a polyphenolic compound or flavonoid. These metabolites were also detected from the crude active EtoAc extracts, as shown in Table 2 and were coded P_1848, N_3284, and N_3285 in the positive and negative mode found at *m*/*z* 517.1341 [M + H]^+^, 515.1196 [M − H]^−^, and 353.0880 [M − H]^−^, respectively. N_14914 was identified as a 2M+ complex of *m*/*z* 515.126 (N_9100), eluting at 8.10 min. While the others did not give any hits and remained unidentified.

## 3. Discussion

### 3.1. Metabolomic-Guided Isolation of Target Anticancer Active Metabolites

The variable features defining the bioactivity of certain fractions in the Jordanian and European varieties were targeted for chromatographic isolation work. They afforded the detection of higher-yielding phenolics in semi-purified bioactive fractions that includes 3,5*-O*-dicaffeoylquinic acid (N_4575) from F8-ChJ, chrysosplenetin (N_435) from F2-ChE, apigenin (N_3180) from F8-ChE, 1,3-*O*-dicaffeoylquinic acid (N_9100) from F11-ChE, and, 4′→1-*O*-feruloylglucose (N_5231)from F12-ChE. Structure elucidation of these known metabolites and their occurrence in respective bioactive fractions were validated by 1D and 2D NMR, and their mass spectral data is described under the Supplementary Information.

The dereplicated secondary metabolites produced by Jordanian and European chamomiles showed similar chemistry, as all analogues belong to phenolics, flavonoids, and quinic acid compounds. The TIC for the active semi-purified fractions shown in Figure 8 indicated the distribution of the various phenolic compounds. Jordanian chamomile yielded 40% of dicaffeoylquinic acid isomers, as exemplified by f8-ChJ (**2**), while European chamomile afforded only 11% of the entire extract, distributed mostly in f11-ChE. Flavonoid compounds were dominantly produced by the European chamomile, with apigenin (**17**) and chrysosplenetin (**36**) as the major flavonoids relatively abundant in f8-ChE and f2-ChE, respectively. In accordance, the proton NMR of the bioactive EtOAc extracts of Jordanian and European varieties (Figure 5a,b) have shown a different chemical profile because of a higher concentration of substituted glycosidic phenolics in European chamomile while there is the occurrence of lower yielding *O*-dicaffeoylquinic acid derivatives in both varieties. The EtOAc extracts from Jordanian chamomile revealed the presence of metabolites with chemical shifts resonating between 6.00 to 7.00 and 9.00 to 12.00 ppm (unsubstituted phenolic compounds), which was an indication of the presence of phenylpropanoids [36]. Meanwhile, the NMR spectral data of European chamomile extracts exhibited a higher density of resonances between 2.0 and 4.0 ppm, indicating the presence of glycosylated or methoxylated and hydroxylated active metabolites, which was further evidence of the presence of flavonoids. However, the antioxidant active *O*-dicaffeoylquinic acid congeners with a chemical formula C_24_H_25_O_24_ found at the ion peak *m*/*z* 517.1341 [M + H]^+,^ 515.1196 [M − H]^−^ was a common target from both varieties eluting at 8.1 and 8.4 min for the European and Jordanian varieties, respectively.

Discriminating metabolites from the Jordanian EtOAc extract yielded medium MW compounds that were derivatives of caffeoylquinic acid, which have been described for their antioxidant activity [29]. These included N_3285 and N_3284, with ion peaks at *m*/*z* 353.0880 and 515.1196 Da. Furthermore, discriminating metabolites with MZmine ID P_7541, P_21550, N_3306, and P_845 with *m*/*z* values of 449.1079, 465.1028, 465.1029, 463.0887, 495.1133 Da were dereplicated as flavonoid derivatives. Numerous flavonoids have been shown to have anticancer properties. However, the molecular mechanisms behind this effect have not been completely understood [30]. The latter metabolites were found in the European chamomile EtOAc extract, which supported the evidence for its anticancer activity against breast cancer cells. A correlation between total phenolic content and antioxidant activity has been earlier reported [31], but not with total flavonoid content.

Ion peaks [M − H]^−^ at *m*/*z* 515.127, and 515.118 Da were pinpointed as the discriminating features for the active EtOAc extracts, and 1031.260 is a 2M^+^ complex of 515.127 Da. Amongst the targeted compounds from the crude EtOAc extract of both varieties, only two compounds could be isolated, F11-ChE and F8-ChJ, present in European and Jordanian chamomile. Both compounds elucidated as 1,3-dicaffeoyl quinic acid and 3,5-dicaffeoyl quinic acid, respectively, have been widely reported for their significant antioxidant activity and scavenging properties [37,38].

However, four of the isolated, targeted discriminating features for the European variety with ion peaks [M − H]^−^ at *m*/*z* 373.093, 269.045, 515.127, and 355.103 Da were associated with the active fractions corresponding to chrysosplenetin, apigenin, 1,3-dicaffeoyl quinic acid and glucoferullic acid, respectively. While one compound was isolated from Jordanian chamomile crude extracts, which was 3,5-dicaffeoyl quinic acid. Both varieties yielded dicaffeoylquinic acid isomers, were pinpointed as the bioactive compound and led to the antioxidant activity of their extracts [37,38]. The ^1^H NMR spectra of F11-ChE were comparable to that of F8-ChJ, which are structural isomers with identical MW, with only a variation in elution time of 0.3 s between them. Even though the spectral data of F11-ChE and F8-ChJ were almost comparable, they were not identical. The compounds are structural isomers with identical MW, varying by 0.3 s in their retention times. Differences were also detected in the respective ^1^H NMR of both compounds. When the ^1^H NMR spectra of F11-ChE and F8-ChJ were compared, it was observed that there was a more obvious separation of the trans olefinic ^1^H signals for the two caffeoyl units. The chemical shift of H3 (4.96 ppm) and H5 (5.11 ppm) was more shifted downfield, which suggested the esterification of two quinic acid moieties at positions 3 and 5. Whereas the chemical shift of the methine proton on C3 was shielded upfield in F11-ChE, it was determined that the caffeoyl unit on 3-OH disappeared, and the esterification of the hydroxyl unit on C1 had taken its place.

In this study, a metabolomics approach applied multivariate analysis in investigating the differences in the production of secondary metabolites of two chamomile varieties grown under different environmental conditions. Changes in metabolite production were perceived between the two varieties. Different environmental conditions between Jordanian and European chamomile affected the type of isolated compounds.

Even though the climate in Jordan varies from a more Mediterranean to a desert environment, the country is typically quite dry. Winter temperatures vary between 9 and 13 °C, and summer temperatures range between 25 and 35 °C [39]. Meanwhile, the climate in the United Kingdom is temperate and oceanic, with more rainfall throughout the year. Seasonal variations in temperature are common; nonetheless, the temperature rarely goes below −10 °C or climbs over 35 °C. In the literature, it was stated that warm and dry weather in *Quercus ilex* leaves increases the concentration of polyphenols [40,41], which explained the production of di-caffeoylquinic acid and underivatized phenolics in higher concentration in Jordanian chamomile.

As a result, significant changes in the synthesis of primary and secondary metabolites were seen between specimens of the same plant species grown under various environmental circumstances. [1,2]. Polyphenols are the most adaptable secondary metabolites, enabling plants to react quickly to various unanticipated stressors, which could be influenced by various environmental factors [7]. The respective category of secondary metabolites, influenced by environmental conditions, serves as a chemical interface between the plant and its environment. These interaction could generate variations in metabolic profiles for the same species [1].

Other factors that affect secondary metabolites productions of the same species appear to be related to the induction of seasonal stress factors in the plant, such as (i) drought stress, which can cause a reduction in photosynthetic rate with a consequent increase in the production of reactive oxygen species (ROS), resulting in an increase in the production of phenolic compounds (natural antioxidants) during the dry season as a defence mechanism [42,43,44]. (ii) Thermal stress caused by a wide temperature variation range throughout the year, particularly during drier periods, which can affect metabolic regulation, permeability to water and CO_2_, as well as the rate of intracellular reactions as well as improve antioxidant properties by increasing the availability of carbohydrates thermal stress [42,45].

### 3.2. Role of Isolated Phenolics and Flavonoids Compound from Chamomile Varieties as Anticancer and Antioxidant Activity

Polyphenols are a class of secondary metabolites found in plants that have been widely investigated. Polyphenols are distinguished by the presence of numerous phenol (benzene) rings in their structure [46]. Flavonoids, phenolic acids, stilbenes, and lignans are all polyphenols. Polyphenols have a wide range of biological roles, including anticancer action, due to their structural diversity [47]. Their basic structures are C6-C3-C6 carbon framework with various substitutions that result in a variety of subclass compounds such as flavones, flavanols, flavanones, isoflavones, flavanols or catechins, and anthocyanins [48].

Flavonoids exert their anticancer effect by inducing apoptosis in cancer cells [30]. However, studies have demonstrated that high flavonoid consumption is related to a lower risk of several forms of cancer. Aside from anticancer action, flavonoid-mediated health advantages include antioxidant activity by eliminating free radicals, which may damage lipids, proteins, and DNA [46,49,50]. In this study, antioxidant and anticancer activity on breast cancer cells were examined, and it was found that chrysosplenetin and apigenin significantly inhibited ZR-75 cells with IC50s of 21.07 and 22.65/mL, respectively, without exerting antioxidant activity. While 3,5-*O*-dicaffeoyl quinic acid, 1,3-*O*-dicaffeoyl quinic acid and glucoferulic acid demonstrated antioxidant activity on DPPH assays with EC_50_s of 0.076, 0.311 and 0.165 µM, no effect on ZR-75 cells was exhibited.

The most prevalent and investigated flavonoid is 4′,5,7-trihydroxyflavone, also known as apigenin, with a molecular mass of 270.24 g/mol. Apigenin is abundant in parsley, celery, chamomile, oranges, thyme, onions, honey, spices, and plant-based drinks [51,52]. Previous studies showed that apigenin inhibits the growth of progesterone-dependent BT-474 breast cancer cells xenograft tumours in nude mice by increasing apoptosis and decreasing HER2/neu expression [53]. It was reported by other studies that apigenin-induced apoptosis in MDA MB-453, SKBR3, and BT-474 breast cancer cell lines [54,55,56]. The observed inhibition of cell proliferation in this HER2-overexpressing in breast cancer was related to a caspase-dependent extrinsic apoptotic mechanism and suppression of the STAT3/VEGF signalling pathway. Furthermore, apigenin showed cytotoxic effects in MCF-7 cells accompanied by morphological changes, reduced motility, decreased intracellular communication due to structural abnormalities and decreased intracellular α-tubulin [57]. The anti-cancer activity of apigenin has been studied extensively in several research as a possible cancer-chemopreventive drug against a diverse range of cancer forms [58].

Apigenin has been demonstrated orally to suppress the metastasis of ovarian cancer cell xenograft tumours in mice, and it has also been proven to suppress the invasion and migration of ovarian cancer cells when administered in vitro. Apigenin’s downregulation of MMP-9 in exposed cells and tissues was attributed to these effects [59].

On the other hand, chrysosplenetin showed antiproliferative effect on the MCF-7 breast cancer cell line With an IC_50_ value of 0.29 µM. It exhibited dual inhibitory activity against topoisomerase I and II. It inhibited topoisomerase II by 83–96% in the 12.5–100 µM range. Molecular docking studies were conducted to understand better chrysosplenetin’s binding mechanism, interactions, and affinity for DNA and topoisomerases I and II. These experiments established that the 4-chromone core and the hydroxyl and methoxy groups are required for intercalation with DNA and inhibition of topoisomerase I and II [60]. Another mechanism underlying its antiproliferative activity is the activation of the mitotic spindle checkpoint through microtubule depolymerisation, which leads to cell apoptosis. The property of microtubule disassembling and their lower toxicity allow them to be considered chemopreventive agents [61].

The phenolics and flavonoids demonstrated outstanding anti-inflammatory and anticancer activities [62,63]. Phenolic compounds, which are plant secondary metabolites, are well-known antioxidants and play an essential role in disease resistance [64]

According to the literature, 1,3-diCQA (1,3-*O*-dicaffeoyl quinic acid) demonstrated significant antioxidant and free radical scavenging properties. The IC_50_ value (40 µM) of 1,3-diCQA was two-fold lower than that of Trolox (positive control), suggesting that 1,3-diCQA had higher antioxidant activity than Trolox [37]. Furthermore, it was stated that 3,5-DiCQA (3,5-*O*-dicaffeoyl quinic acid) demonstrated significant antioxidant activity, which assayed on DPPH with an IC_50_ of 4.26 µg/mL, ABTS (2,2′-azino-bis(3-ethylbenzothiazoline-6-sulfonic acid) radical scavenging assay with TEAC (Trolox equivalent antioxidant capacity) value of 0.9974, and FRAP (Ferric reducing antioxidant power) activity with 3.84 mmole of Trolox equivalent/g activities [38]. Also, *Z*-glucoferulic acid has also been reported in the literature to exhibit antioxidant activity [65].

## 4. Materials and Methods

### 4.1. Materials and Samples

#### 4.1.1. Plant Material and Preparation of Extracts and Fractions

**Figure 12:**
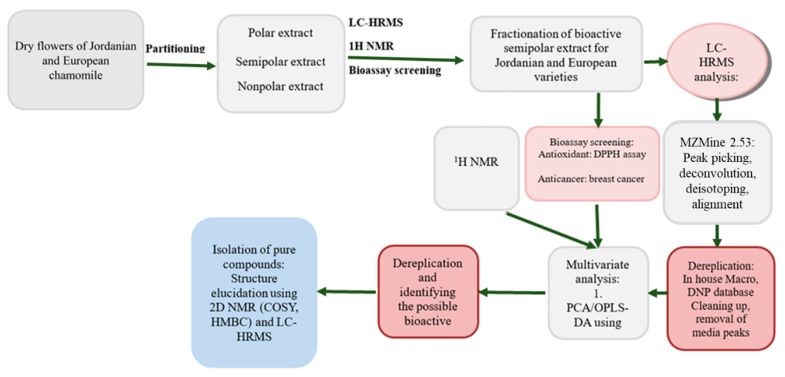
The workflow for isolating the bioactive metabolites from European and Jordanian chamomile.

Jordanian *Matricaria chamomilla* plants used in this study were collected from Bereen Amman, Jordan. The identity of this sample was confirmed by Keith Watson, botanist curator of Kelvingrove Museum in Glasgow. European variety was purchased on Neals Yard Remedies Direct, Glasgow. Both plant samples were treated following the same extraction procedure, 200 g of plant tissue, mainly flowers, were milled and ground. The obtained powder was macerated and stirred in 1000 mL of acetone for 3 h after the mixture was soaked overnight, filtered, and the supernatant and filtrate were collected. This extraction process was done three times. The solid residue was extracted again following the same procedure using methanol instead of acetone. All liquid fractions were pooled together and dried under reduced pressure. Total crude was reconstituted in 200 mL of (90:10) methanol-water solution and sonicated for 5 min at 40 °C. The polar solution was added to a separatory funnel with equal volumes of hexane and extracted three times. The nonpolar phase was collected and similarly dried under reduced pressure. Methanol was evaporated from the polar phase and then reconstituted in 200 mL of water. The aqueous solution was added to a separatory funnel, and the solvent was extracted with EtoAc three times. Both extracts were dried, obtaining the semi-polar and polar fractions. Metabolomics workflow for Jordanian and European chamomile is presented below in Figure 12.

Various analytical and HPLC grade solvents were used for multipurpose procedures that include plant extraction, solvent partitioning, LC-HRMS and MPLC; HPLC-grade acetonitrile (ACN) with 0.01% formic acid both from Sigma-Aldrich, Poznań, Poland was used. HPLC-grade dichloromethane (DCM), n-Hexane, acetone, and methanol (MeOH) from Sigma-Aldrich, Poznań, Poland, were purchased. While analytical-grade acetone from VWR, Fontenay-sous-Bois Cede, France and HPLC-grade ethyl acetate (EtOAc) from Sigma-Aldrich, Poznań, Poland, were used as extraction solvents.

#### 4.1.2. Data Processing and Chemical Profiling

Data were processed with “Xcalibur 2.2”, released by Thermo Scientific, Heidelberg, Germany. In addition, data splitting was done with ProteoWizard (https://sourceforge.net/projects/proteowizard, accessed on 3 January 2019), USA, to separate the data between the negative and positive ionization files. While split data analysis was done with MZmine 2.4.2 followed by an in-house Excel sheet called Macro coupled with the Dictionary of Natural Products (DNP version 2021) database published by CRC Press, Boca Raton, USA [26]. The NMR samples have been dissolved using deuterated dimethyl sulfoxide (DMSO-*d*_6_), methanol (CD_3_OD) and deuterium water (D_2_O) purchased from Sigma-Aldrich, Poznań, Poland and transferred to NMR tubes obtained from Norell, New York, NY, USA. ^1^H NMR spectral data were measured in a 400 or 500MHz Bruker spectrometer (Bruker, Bremen, Germany) and then processed using MNova 2.14 software (Mestrelab, Santiago de Compostela, Spain).

#### 4.1.3. Biological Assay

Normal fibroblasts derived from human foreskin (HS27) cells and human Caucasian breast carcinoma (ZR-75) cells were obtained from ECACC (Sigma-Aldrich, Dorset, UK). HS27 cells were cultured in Dulbecco’s Modified Eagle’s Medium (DMEM), and ZR-75 cells were cultured in RPMI 1640 media; both were supplemented with 10% (*v*/*v*) foetal bovine serum, 2 mM glutamine and 50 µg/mL penicillin/streptomycin solution (all Invitrogen, Paisley, UK) in a humidified incubator at 37 °C in the presence of 5% CO_2_. TrypLE Express was procured from GIBCO, UK. Hanks’ balanced salt solution (HBS) and Trytox X-100 were brought from Sigma, US. Stock solution and samples were solubilised in Dimethyl Sulfoxide (DMSO) was obtained by Sigma-Aldrich (St. Louis, MO, USA).

Cells were routinely passaged at 90–95% confluence. Subsequently, cells were seeded at a concentration of 7500 (HS27) or 3750 (ZR-75) cells/well in clear 96 flat-bottomed plates and allowed to adhere overnight. Afterwards, metabolite extracts were prepared at a stock concentration of 1 mg/mL, added at a final concentration of 30 µg/mL, and allowed to incubate for 42 h. Viability was determined using Alamar Blue^®^ (Thermo Fisher, Paisley, UK), according to the manufacturer’s instructions and incubated for a further 6 h. The resulting fluorescence was measured using a Wallac Victor 2 1420 multi-label counter (Perkin Elmer, Beaconsfield, UK) in fluorescence mode: excitation 560, emission 590. Vehicle-treated control cells (media with 0.3% DMSO) were considered 100% viable against which metabolite extract treated cells (at a concentration of 30 µg/mL, at least n = 3) were compared. On the 96-well plate, 75 µL of the cell culture was added. As a positive control, 25 µL of media was added to the cells after they were treated with 30 µg/mL of the plant extract. As a negative control, 4% of TrytonX was used. All results were confirmed microscopically.

% Inhibition = (Test or standard compound RFU-Background/Control RFU-Background) × 100. Data were then expressed as mean ± SEM and were plotted using Graph Pad Prism Software (Graph Pad Prism version 6.00 for Windows, GraphPad Software, La Jolla, CA, USA, www.graphpad.com, accessed on 1 May 2020). The threshold of activity for samples was set at 60% inhibition (40% of the control)

Table 6 illustrates the seeding density for the cell lines utilised in a flask and plate.

For IC_50_ calculation, serial dilution was prepared to have 8 points of concentration (100, 30, 10, 3, 1. 0.3, 0.1, 0.03, 0.01) in µg/mL for each fraction in DMSO for Jordanian and European chamomile fractions. Both cells were seeded in 96 flat-bottomed plates at a concentration of 7500 (HS27) or 3750 (ZR-75) cells/well and allowed to adhere overnight. The IC_50_ value was determined using AlamarBlue. Alamar blue was applied to the wells at a concentration of 10% and incubated for 6 h. Fluorescence was measured, and the Alamar Blue test was conducted three times. The data were analysed using Excel and GraphPad Prism 6.

Curve fitting was performed to calculate the IC_50_ using the following sigmoidal dose-response formula: Y = bottom + (top-bottom)/{1 + 10^(Log IC50 − X)^} in GraphPad prism 6.

#### 4.1.4. Antioxidant Activity

2,2-diphenyl-1-picrylhydrazyl (DPPH), ascorbic acid and quercetin were purchased from Sigma-Aldrich, Poznań, Poland.

The presence of antioxidant compounds in the plant extracts was assessed using a DPPH assay. The antioxidant potential of the different polar fractions was estimated from their capacity to reduce free radical DPPH (2,2-diphenyl-1-picryl-hydrazyl-hydrate) [66]. The redox reaction produces a violet colour in the presence of ethanol that can be measured at a wavelength of 520 nm. Stock solutions of 1 mg/mL of semi-polar and nonpolar plant extracts were prepared and dissolved in DMSO, while the aqueous extract was in distilled water. An aliquot of 1, 0.5, 0.25, 0.125, 0. 0625, 0.03125 and 0.007813 mg/mL were transferred to a 96-well plate in triplicate. The antioxidant activity was tested by the addition of 0.6 mM DPPH solution. A solution of 0.6 mM DPPH in absolute ethanol was used as a control. The assay background was determined by only measuring the optical density of DMSO and DPPH reagent. The reaction was left to develop in the dark at room temperature (25 °C) for 30 min. The absorbance was measured at 520 nm, and the percentage of inhibition was calculated by the following equation.

% Antioxidant activity = OD control − OD sample/OD control 100%

Plotted dose-response curve between and % of antioxidant activity vs. Concentration was performed, and linear regression analysis was done to calculate the effective concentration to scavenge DPPH free radical at 50% (EC_50_).

### 4.2. Fractionation of Active Extracts

Bioactive EtOAc extracts from both chamomile varieties were fractionated employing the same solvent gradient. Semi-automated flash chromatography was carried out using a two-pump Buchi Sepacore^®^ system. A gram of each of the Jordanian European extracts was respectively loaded in celite and eluted over a Versa Flash Silica cartridge column 40 × 150 mm. The chromatographic protocol was performed using a solvent system of A: MeOH and B: DCM with a 100 mL/min flow rate. The gradient used was 20 min at 99% of B, then 10 min at 98% of B, 10 min at 97% of B, 6 min at 95% of B, 6 min at 90% of B, 4 min at 80% of B, and the column was washed for 8 min with 70% acetone and 30% of MeOH. The fraction collection volume was set at 10 mL/tube. TLC was carried out to display the separation profiles of the fractions and similar fractions was pooled together. The pooled fractions were concentrated under vacuum by a rotary evaporator to give 12 major fractions from the European chamomile extract and eight major fractions from the Jordanian variety. The resulting fractions were analysed using NMR and HRESI-MS for metabolomic studies and tested for antioxidant and anti-cancer activity.

### 4.3. Metabolomic Profiling Studies

#### 4.3.1. Acquisition of Analytical Data NMR and HRESI-MS

Raw extracts obtained were analysed by NMR and mass spectroscopy techniques for a first approach to the type of compounds present in each sample. For NMR analysis, nonpolar and semipolar samples were dissolved in 600 µL deuterated dimethyl sulfoxide, and the aqueous samples were dissolved in 600 µL deuterium water. Then all the samples were transferred to NMR tubes at a concentration of 5 mg/mL. The acquisition of NMR ^1^H one-dimensional and ^1^H COSY bidimensional spectra was recorded on a 400 MHz Jeol instrument.

For HRESI-MS experiments, HPLC-grade MeOH and acetonitrile (ACN) were used to dissolve the nonpolar and semipolar samples, while HPLC-grade distilled water was used for the aqueous samples in a concentration of 1 mg/mL. The acquisition of HRESI-MS chromatograms was performed on an Accela HPLC Thermo Scientific, coupled with a UV detector at 280 and 360 nm and an Exactive-Orbitrap high-resolution mass spectrometer Thermo Scientific. The samples were eluted through a C-18 column (ACE, 75 mm, id 3.0 mm, particle size 5 μm). All runs were operated under 37 bar pressure and 22 °C. Thermo Xcalibur 2.1 software (Thermo Fisher Scientific, San José, CA, USA) was used to operate the process, and the MassConvert was used to split the raw data to separate positive and negative ionisation files that will be imported to MZmine 2.53. Then the data was then analysed using SIMCA 17. The same methodology was used for the pure compounds obtained after the fractionation process.

#### 4.3.2. HRESI-MS Data Process for Dereplication and Multivariate Analysis

Raw data were initially divided into two sets based on positive and negative ionization modes, using the MassConvert tool from ProteoWizard. Those sliced data sets were subsequently loaded and processed in MZmine 2.53. The data process was performed following these treatment steps: mass detection (Mass detector: Centroid, Noise level: 1000, MS level: 1), chromatogram builder (Min time span (min): 0.2, Min height: 1 × 10^4^, *m*/*z* tolerance: 0.001 *m*/*z* or 5 ppm), deconvolution (Chromatographic threshold: 5%, Search minimum in RT range (min): 0.4, Minimum relative height: 5%, Minimum absolute height: 10000, Min ratio of peak top/edge: 3, Peak duration range (min): 0.2–5), deisotoping (*m*/*z* tolerance: 0.001 *m*/*z* or 5 ppm, Retention time tolerance: 0.1 absolute (min), Maximum charge: 2, Representative isotope: Most intense), filtering (Retention time range 5 40 min.), alignment (*m*/*z* tolerance: 0.001 *m*/*z* to 5 ppm, Weight for *m*/*z*: 20, Retention time tolerance: 5%, Weight for RT: 20) and gap filling (*m*/*z* tolerance: 0.001 *m*/*z* or 5 ppm, Intensity tolerance: 30%). The prediction steps include the adducts identification (RT tolerance: 0.2 absolute (min), Adducts: Na^+^, K^+^, NH_4_^+^ for positive mode and HCOO^−^ for negative mode and CH_3_CN+H for both modes, *m*/*z* tolerance: 0.001 *m*/*z* or 5 ppm, Max relative adduct peak height: 30%), identification of complex (Ionization method: [M + H]^+^ for positive mode and [M − H]^−^ for negative mode, Retention time tolerance: 0.2 absolute (min), *m*/*z* tolerance: 0.001 *m*/*z* or 5 ppm, Max complex peak height: 50%) and formula prediction (Select ionization mode, either [M + H]^+^ or [M − H]^−^, Isotopic *m*/*z* tolerance: 0.001 or 5.0 ppm, Minimum absolute intensity: 5.0 × 10^−5^, Minimum score: 65%). Mass data was then exported as a CSV file (comma delimited) for further clean-up. [66] Negative and positive datasets were imported to the macro removing all blanks from media and samples. This step prepared the data set for multivariable data analysis in SIMCA 17. The dereplication stage was performed using the DNP database.

#### 4.3.3. NMR Data Process for Multivariate Analysis (MVA)

NMR spectral data were processed using MNova 2.14 software (Mestrelab, Compostela, Spain). Once the data were loaded in the software, they were Fourier-transformed, phased and baseline corrected. All sample spectra were stacked and binned at a spectral width of 0.04 ppm. The resulting file was copied into an Excel file in a CSV format for MVA treatment.

#### 4.3.4. Multivariate Analysis

MVA data were processed on SIMCA 17. The scaling applied was Pareto. The metabolic profiles of different samples were compared using PCA and OPLS-DA models to evaluate their unique metabolites fingerprint. The validations of both models were discriminated by a Q^2^ and R^2^ permutation test. The absence of overfitting was measured by the difference of Q^2^ and R^2^Y values, which must be less than 0.3.

## 5. Conclusions

The study aimed to investigate the differences in the production of secondary metabolites of two chamomile varieties grown under different environmental conditions. The Jordanian chamomile was collected with exact geographical information; however, no precise collection location could be obtained for the European chamomile. In Jordan, chamomile is only grown in gardens for individual use and not cultivated for commercial applications. Unlike the European chamomile, which has been widely cultivated in such countries as Germany and UK and sold commercially as a herbal remedy for alternative medicine. *M. chamomilla* is one of the traditional folk medicinal plants used for centuries. It treated several diseases, including stomachache, flatulence, carminative, gingivitis, diuretic, anti-inflammatory, aromatic, and acute respiratory infections. European and Jordanian chamomile were obtained from the same species but in different geographical locations. Different chemical profiles and biological activities were expected and confirmed after their similar NMR, HR-LC/MS data analysis, and biological results.

After fractionation of EtOAc of two chamomile varieties, both European and Jordanian chamomile fractions produced dicaffeoyl quinic acid isomers that exhibited an antioxidant activity with EC_50_ values of 0.311 µg/mL for F11-ChE and 0.076 µg/mL for F8-ChJ. However, the Jordanian variant exhibited the most potent antioxidant activity because dicaffeoyl quinic acid was a significant compound in higher concentrations. This was rationalised due to Jordan’s warm and dry weather, which increased the concentration of underivatized polyphenols [40].

Nevertheless, it was clear that the European chamomile was producing more classes of metabolites, as shown by the metabolomic data analysis. The isolated target compounds were all known compounds. The results revealed that chrysosplenetin (F2-ChE) and apigenin (F8-ChE) compounds from European fractions had anticancer activity against ZR-75 cells with IC_50_ values of 21.07 and 22.55 µg/mL, respectively. Those compounds were also assayed for their toxicity against human foreskin fibroblast cells (HS-27), and both were not toxic and, therefore, selective for the triple negative cancer cell line-ZR75. In addition, glucoferulic acid (F12-ChE) from European chamomile displayed antioxidant activity with EC_50_ values equal to 0.165 µg/mL. Changes in metabolite production were perceived between the two varieties.

### Final Conclusions

There are differences in the production of metabolites between the Jordanian and European varieties of chamomile. These environmental conditions determined the type of the isolated compounds and, subsequently, their efficacy against triple-negative breast cancer cells while showing no effect against normal human cells.

## Figures and Tables

**Table 1:** EC_50_ values of antioxidant activity assay of liquid-liquid partitioned extracts obtained from both varieties of *M. chamomilla* in mg/mL. Ascorbic acid and quercetin were used as positive controls.

Type of Extract	Jordanian Chamomile (mg/mL)	European Chamomile (mg/mL)	n
n-Hexane	inactive	inactive	3
10% aq MeOH	inactive	inactive	3
EtOAc (Mean ± SEM)	0.20 (0.20 ± 3.29 × 10^−5^)	0.16 (0.16 ± 2.77 × 10^−5^)	3
ascorbic acid (control) mg/mL (Mean ± SEM)	0.11 (0.11 ± 1.43 × 10^−5^)		3
quercetin (control) mg/mL (Mean ± SEM)	0.03 (0.03 ± 4.00 × 10^−4^)		3

n = number of samples, SEM = standard error of mean.

**Table 2:** Dereplication of the discriminating metabolites with *p* < 0.05 for outliers determined from the OPLS-DA loadings plot (Figure 3b) of the bioactive metabolite from either variety of *M. chamomilla* EtOAc extracts. A highlighted row represents a compound isolated from the active EtOAc extracts. Metabolites were listed according to increasing *p*-values. Compound hits were filtered to those belonging to the family Asteraceae except for those limited only to one or two hits. Compound structures are shown in Figure 4.

Var ID *	*p*-Values	*m*/*z* (Da)	Rt (min)	MW	Name	Molecular Formula	Reported Biological Source in the Database
P_1931	3.60 × 10^−6^	163.0389	7.84	162.0317	7-hydroxy-2*H*-1-benzopyran-2-one (**1**)	C_9_H_6_O_3_	*M. chamomilla*
N_3284	5.31 × 10^−5^	515.1196	8.43	516.1269	7″-*Z*-3,5-di-*O*-caffeoylquinic acid (**2**) ** and positional isomers	C_25_H_24_O_12_	*Arnica montana*
P_1848	2.16 × 10^−4^	517.1341	8.43	516.1269	3″,4″-di-acetylcosmosiin (**3**) **	C_25_H_24_O_12_	*M. chamomilla*
					*O*-[3,4-dihydroxy-*E*-cinnamoyl-4-α-d-glucopyranoside] (**4**) **		*Erycibe obtusifolia*
P_21550	2.51 × 10^−4^	465.1029	7.55	464.0956	2′,4′,5,5′,7 pentahydroxyflavone (**5**) **	C_21_H_20_O_12_	*Artemisia hispanica*
P_7541	2.51 × 10^−4^	465.1028	7.55	464.0956	7-*O*-β-d-glucuronopyranoside (**6**) **	C_21_H_20_O_12_	*Chrysanthemum indicum*
N_3306	1.36 × 10^−3^	463.0887	7.61	464.0960	bracteoside (**7**)	C_22_H_22_O_12_	*Centaurea bracteatassssss*
P_1044	2.83 × 10^−3^	499.1235	8.46	498.1162	elenoside (**8**)	C_25_H_22_O_11_	*Justicia hyssopifolia* (no hits found from the f. Asteraceae)
N_3290	5.26 × 10^−3^	493.0991	7.72	494.1064	3-*O*-methylgossypetin 7-glucoside (**9**) **	C_22_H_22_O_13_	*Artemisia fragrans*
N_3285	1.06 × 10^−2^	353.0880	8.34	354.0952	5-*O*-*E*-caffeoylquinic acid (**10**)	C_16_H_18_O_9_	*Cynara scolymus* *Cydonia oblonga,* *Aster scabe*
P_845	1.50 × 10^−2^	495.1133	7.76	494.1061	3,4′,5,6,7 pentahydroxy 3′-methoxyflavone (**11**) **	C_22_H_22_O_13_	*Eupatorium tinifolium*
P_716	4.06 × 10^−2^	787.3694	12.56	786.3622	*N*^1^,*N*^5^,*N*^10^,*N*^14^- tetra-trans-p-coumaroylspermine (**12**)	C_46_H_50_N_4_O_8_	*M. chamomilla*

* The letters P and N represents the ionization mode. ** Respectively, compound hits can be interchangeable for identical molecular formulae.

**Table 3:** Dereplication of discriminatory and bioactive target bioactive metabolites against breast cancer (ZR-75) and antioxidant activity as predicted by OPLS-DA loadings plot shown in Figure 9D. Metabolites were arranged according to their ascending *p*-values. Compound hits were filtered to those belonging to the family Asteraceae except for those limited only to one or two hits. Highlighted rows represent compounds validated from semi-purified active fractions of either chamomile varieties investigated in this study and pointed out in Figure 8. Compound structures are shown in Figure 7.

Primary ID *	*p*-Value	*m*/*z*	Rt (min)	MW	Compound Hits	Molecular Formula	Source
N_5225	0.0020	355.1031	5.54	356.1104	*Z*-glucoferulic acid/4′→1-*O*-feruloylglucose (**13**)	C_16_H_20_O_9_	*Equisetum arvense*
					4′-*O*-β-d-galactopyranoside (**14**)		
					2′-hydroxy, 4′-Me ether,1-*O*-p-coumaroyl glucose (**15**)		*M. chamomilla*
					4′-*O*-β-d-allopyranoside (**16**)		*Cimicifuga dahurica* *C. heracleifolia*
N_3180	0.0137	269.0452	12.55	270.0525	apigenin (**17**)	C_15_H_10_O_5_	wide range of plant spp.
					2-(2,4-dihydroxyphenyl)-5,6-methylene dioxybenzofuran (**18**)		*Artemisia indica*
N_11757	0.0144	655.4065	13.55	656.4137	bractic acid (**19**)	C_35_H_60_O_11_	*Ajuga bracteosa*
					orbicoside (**20**)		*Astragalus orbiculatus*
N_2695	0.0145	785.3552	12.92	786.3625	see Compound **12**	C_46_H_50_N_4_O_8_	*M. chamomilla*
N_11748	0.0146	445.2447	13.37	446.2520	2-acetyl-3-*O*-(2-methylpropanoyl) β-d-glucose-4-*O*-(8-methylnonanoyl) (**21**)	C_22_H_38_O_9_	*Solanum aethiopsicum* (only 2 hits)
N_9100	0.0146	515.1190	8.10	516.126	see compound **2**	C_25_H_24_O_12_	
N_11749	0.0165	971.4864	14.43	972.4937	calendasaponin B (**22**)	C_48_H_76_O_20_	*Calendula officinalis*
N_11744	0.0165	447.0921	7.68	448.0994	carthamone (**23**) luteolin 6-*C*-glucoside (**24**)	C_21_H_20_O_11_	*Carthamus tinctorius* *Artemisia princeps*
N_3234	0.0169	329.2329	12.89	330.2402	pinellic acid (**25**)	C_18_H_34_O_5_	*Helianthus heterophyllus*
N_11753	0.0188	441.2497	14.53	442.2570	*O*-(2-*O*-acetyl-β-d-fucopyranoside)-11- hydroxy-4,5-secoeudesmane-4,5-dione (**26**)	C_23_H_38_O_8_	*Carthamus arborescens*
N_5224	0.0202	655.1293	9.14	656.1366	tinctoside (**27**)	C_31_H_28_O_16_	*Anthemis tinctoria*
N_11652	0.0236	493.0977	7.79	494.1049	see compounds **9** and **11**	C_22_H_22_O_13_	
N_5231	0.0421	355.1026	7.23	356.1098	see N_5225	C_16_H_20_O_9_	see N_5225
N_5236	0.0515	463.0874	7.56	464.0947	see compounds **5** and **6**	C_21_H_20_O_12_	
N_5226	0.0653	711.2130	7.23	712.2203	theaflavanoside II (**28**)	C_32_H_40_O_18_	*Camellia sinensis*
N_3484	0.0670	353.0872	8.85	354.0944	see Compound **9**	C_16_H_18_O_9_	
N_4575	0.0772	515.1182	8.47	516.1255	a structural isomer of N_9100	C_25_H_24_O_12_	
N_11743	0.0868	516.1220	8.29	517.1290	No hits	-	*-*
N_2900	0.1009	431.0976	8.61	432.1049	thalictiin (**29**)	C_21_H_20_O_10_	*Thalictrum thunbergia* *Chrysanthemum* *morifolium*
N_5222	0.10510	1031.2400	8.35	1032.2500	No hits	not predicted	*-*
N_14912	0.1085	515.1265	8.09	516.1338	methyl ester of antibiotic a 503083f (**30**)	C_19_H_24_N_4_O_13_	*Streptomyces sp. SANK 62,799* (only 1 hit)
N_14913	0.1085	711.2250	7.23	712.2320	No hits	not predicted	*-*
N_14914	0.1085	1031.2606	8.10	1032.2679	[2M − H] of *m*/*z* 515.126 (N_9100)		*-*
N_2089	0.1104	353.0930	8.48	354.0993	No hits	not predicted	*-*
N_2649	0.1406	359.0772	13.57	360.0845	7,8′-cyclo-3′,4,4′,5-tetrahydroxy-2,7′-lignan-9,9′-dioic acid di-Me ester (**31**) 5,7-dihydroxy-3′,4′,6-trimethoxyflavonol (**32**) sudachitin (**33**) quercetagetin 3,3′,6-trimethyl ether (**34**) chrysosplenol D (**35**)	C_18_H_16_O_8_	*Helianthus annuus* *Arnica chamissonis* *Artemisia klotzchiana* *Centaurea hyssopifolia, * *Tanacetum parthenium* from many Asteraceae
N_435	0.3460	373.0926	15.56	374.0999	chrysosplenetin (**36)**	C_19_H_18_O_8_	from many Asteraceae

* The letters P and N represent the ionization mode. Targeted bioactive metabolites were only ionizable in the negative mode.

**Table 4:** EC_50_ for the bioactive fractions that possess antioxidant activity.

Samples	Trend Line Equation	R^2^	EC_50_ mg/mL	Mean ± SEM	n
F11 ChE	y = 15.466ln(x) + 68.738	0.9156	0.311	0.311 ± 2.21 × 10^−5^	3
F12 ChE	y = 11.573ln(x) + 67.599	0.9249	0.165	0.165 ± 1.20 × 10^−5^	3
F8 ChJ	y = 9.3158ln(x) + 74.126	0.9605	0.076	0.076 ± 2.61 × 10^−5^	3
ascorbic acid	y = 10.592ln(x) + 70.222	0.9664	0.146	0.0146 ± 4.63 × 10^−5^	3
quercetin	y = 16.534ln(x) + 104.2	0.9383	0.034	0.034 ± 4.60 × 10^−4^	3

n = number of samples, EC_50_ = half maximal effective concentration, SEM = standard error of mean.

**Table 5:** IC_50_ values in μg/mL for the bioactive European chamomile fractions against the tested cell lines. If SI greater than 3 or the IC_50_ of the tested normal cell was not obtainable, then the compound is considered selective [35]. SI = selectivity index, SEM = standard error of the mean.

Bioactive Fraction	IC_50_ ZR-75 (µg/mL)	IC_50_ HS-27 (µg/mL)	Toxicity	SI
F2-ChE (Mean ± SEM)	21.07 (21.07 ± 5.31 × 10^−6^)	>100 (2.60 × 10^−7^)	Not toxic	>4.74 selective
F8-ChE (Mean ± SEM)	22.65 (22.65 ± 6.30 × 10^−6^)	>100 (1.88 × 10^−7^)	Not toxic	>4.41 selective

**Table 6:** Seeding densities (cell/cm^2^) for the used cell lines.

Cell Line	Flask	96 Well Plates
**ZR-75**	8 × 10^3^	1 × 10^5^
**HS-27**	2 × 10^4^	1 × 10^5^

## Data Availability

Not applicable.

## Supplementary Materials

The following supporting information can be downloaded at: https://www.mdpi.com/article/10.3390/plants12122297/s1, Table S1: ^1^H and ^13^C NMR data of F8-ChJ in CD_3_OD, Table S2: ^1^H and ^13^C NMR data of F2-ChE in comparison to chrysosplenetin (Alwahsh et al., 2015), Table S3: ^1^H NMR data of F8-ChE and synthesised apigenin measured at 400 MHz, Table S4: ^1^H NMR data(δ_H_ in ppm, mult, *J* in Hz) of F11-ChE in comparison to cynarin, a 1,3-dicaffeoylquinic acid analogue along with spectral data of 1,5-dicaffeoylquinic acid reported in the literature, Table S5: ^1^H NMR data (δ_H_ in ppm, mult, *J* in Hz) of F12-ChE in comparison to that of 1,3-*O*-dicaffeoylquinic acid (F11-ChE) and 3,5-*O*-dicaffeoylquinic acid (F8-ChJ) measured at 400 MHz in DMSO-*d*_6_, Table S6: ^1^H NMR data (δH in ppm, mult, J in Hz) of F12-ChE in comparison to that of 4′→1-*O*-feruloyl-β-d-glucose and 1-*O*-feruloyl-β-d-glucose as reported in the literature. Figure S1: Chemical structure of F8-ChJ, 3,5-*O*-dicaffeoylquinic acid. Chemical Formula: C_25_H_24_O_12_, Exact Mass: 516.1264, Figure S2: Extracted ion chromatogram and mass spectrum of F8-ChJ from the Jordanian chamomile showing source fragmentation at *m*/*z* 515.1182 [M − H]^−^ eluting at 8.47 min, Figure S3: Fragment scheme of a dicaffeoylquinic acid isomer. Figure S4: ^1^H NMR spectrum of F8-ChJ in CD_3_OD measured at 400 MHz, Figure S5: (^1^H-^1^H) COSY correlation spectrum of F8-ChJ in CD_3_OD measured at 400 MHz, Figure S6: HMBC spectrum of F8-ChJ in CD_3_OD measured at 400 MHz, Figure S7: Chemical structure of F2-ChE, chrysosplenetin, Chemical Formula: C_19_H_18_O_8_, MW: 374.1000, Figure S8: Extracted ion chromatogram of F2-ChE for the ion peak at *m*/*z* 375.1080 [M + H]^+^ at a RT of 15.5 min, Figure S9: Extracted ion chromatogram of F2-ChE for the ion peak at *m*/*z* 373.0927 [M − H]^−^ at a RT of 15.5 min, Figure S10: ^1^H NMR spectrum of F2-ChE in DMSO-*d*_6_ measured at 400 MHz, Figure S11: HMBC spectrum of F2-ChE in DMSO-*d*_6_ measured at 500 MHz, Figure S12: Chemical structure of F8-ChE, apigenin, Chemical Formula: C_15_H_10_O_5_, Exact Mass: 270.0525, Figure S13: Extracted ion chromatogram of F8-ChE for the ion peak at *m*/*z* 269.0450 [M − H]^−^ eluting at RT of 12.5min Figure S14: Extracted ion chromatogram of F8-ChE for the ion peak at *m*/*z* 271.0600 [M + H]^+^ eluting at RT of 12.5 min, Figure S15: 1H NMR spectrum of F8-ChE in DMSO-*d*_6_ measured at 400 MHz, Figure S16: Chemical structure of F11-ChE, Chemical Formula: C_25_H_24_O_12_, MW: 516.1261, Figure S17. Extracted ion chromatogram of F11-ChE for the ion peak at *m*/*z* 517.1338 [M + H]^+^ with an RT of 8.1 min, Figure S18. Extracted ion chromatogram of F11-ChE for the ion peak at *m*/*z* 515.1117 [M − H]^−^ with an RT of 8.1 min, Figure S19: ^1^H NMR spectrum of F11-ChE in DMSO-*d*_6_ measured at 400 MHz, Figure S20: Isopropylidene derivatisation of 1,3-*O*-dicaffeoylquinic acid, Figure S21: COSY NMR spectrum of the quinic acid moiety in F11-ChE measured in DMSO-*d*_6_ at 400 MHz, Figure S22: Chemical structure of F12-ChE, 4′→1-*O*-feruloylglucose, chemical Formula: C_16_H_20_O_9_ MW: 356.1179, Figure S23: Extracted ion chromatogram of F12-ChE for the ion peak at *m*/*z* 355.1030 [M − H] eluting at an Rt of 7.23 min, Figure S24: Extracted ion chromatogram of F12-ChE for the ion peak at *m*/*z* 357.1180 [M + H] eluting at an Rt of 7.24 min, Figure S25: Source fragmentation of the ion peak at *m*/*z* 357.1180 of F12-ChE, Figure S26: ^1^H NMR spectrum of F12-ChE in DMSO-*d*_6_ measured at 400 MHz, Figure S26: 1H NMR spectrum of F12-ChE in DMSO-d6 measured at 400 MHz, Figure S27: COSY NMR spectrum F12-ChE measured in DMSO-*d*_6_ at 400 MHz. The blue line represents the propenoic acid moiety, the yellow line is the ABX system of the 1,3,4-trisubstituted phenyl system of the ferulic acid unit, and the green line traces the coupling protons for the glucose unit.
