# Supplementary material for: Use of Multivariate Analysis to Unravel the Differences between Two Chamomile Varieties and Their Anticancer and Antioxidant Activities
Source: Plants (Basel). 2023 Jun 12;12(12):2297. doi: 10.3390/plants12122297 (PMC10304069; doi:10.3390/plants12122297)
Supplement: Supplementary file 1 [file plants-12-02297-s001.zip › plants-2373747-supplementary.pdf]

Use multivariate analysis to unravel the differences between two Chamomile varieties and their anticancer and antioxidant activities.

Dana Atoum [1,2], Ignacio Fernandez-Pastor [1,3], Louise Young [1] and, Ruangelie Edrada-Ebel [1*]

[1] Strathclyde Institute of Pharmacy and Biomedical Sciences, University of Strathclyde, Glasgow G4 0RE, UK. ruangelie.edrada-ebel@strath.ac.uk (R.E.-E.); louise.c.young@strath.ac.uk (L.Y.)
[2] Department of Pharmaceutical Chemistry, Faculty of Pharmaceutical Sciences, The Hashemite University, Zarqa, Jordan, dana.atoum@hu.edu.jo (D.A.)
[3] Fundación MEDINA, Avenida del Conocimiento, 34, 18016, Armilla, Granada, Spain. ignaciofernandezpastor@gmail.com (I.F-P.)

* Strathclyde Institute of Pharmacy and Biomedical Sciences, University of Strathclyde, Glasgow G4 0RE, UK.
ruangelie.edrada-ebel@strath.ac.uk
* Correspondence: ruangelie.edrada-ebel@strath.ac.uk

1 Structural elucidation of biological active fractions in Jordanian chamomile

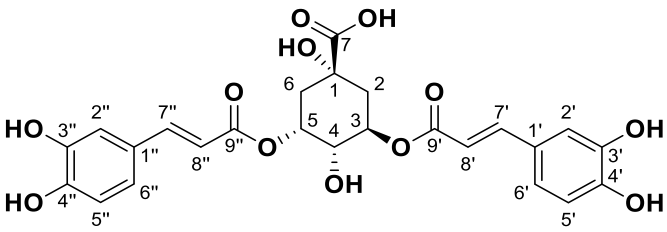

Figure S1. Chemical structure of F8-ChJ, 3,5-*O*-dicaffeoylquinic acid. Chemical Formula: $C_{25}H_{24}O_{12}$, Exact Mass: 516.1264.

The total ion chromatogram of the bioactive fraction F8-ChJ (Figure S2) presented a major compound with following ions peaks at *m/z* 179.0341 for $C_9H_7O_4$, 191.0552 for $C_7H_{11}O_6$, 353.0879 for $C_{16}H_{17}O_9$, 515.1187 for $C_{25}H_{23}O_{12}$ $[M{-}H]^-$, and 1031.2450 for $[2M{-}H]^-$ $C_{50}H_{47}O_{24}$ with a RT of 8.47 min and average intensity of 4.0E+7 (Figure S2). The fragmentation pathway of the spectral data was comparable to those reported in the literature. The MS fragmentation of F8-ChJ is compatible to that of a dicaffeoylquinic acid isomer (Figure S3). The structure of F8-ChJ was presented in Figure S1. Fraction F8-ChJ was 30% of a gram of the crude extract affording 300 mg of 60% 3,5-*O*-dicaffeoylquinic acid as indicated by its TIC in Figure 8 and ^{1}H nmr shown in Figure S4.

F8-ChJ was isolated as a yellow amorphous powder with a yield of 299 mg. HREIMS analysis showed molecular ion peak at *m/z* 515.134 $[M{-}H]^-$, corresponding to the molecular formula $C_{25}H_{23}O_{12}$. The ^{1}H NMR spectrum in Figure S4 revealed the presence of three oxygenated methine protons at δH 5.36 (1H, t, J=6.0, H3), 5.86 (1H, d, J=13.1, H5), and 4.94 (1H, dd, J = 13.0, 6.0 Hz, H4), two methylenes at δ_H 2.30 (1H, q, J =5.7 Hz, H6eq), 2.12 (1H, m, H6ax), 2.09 (1H, br s, H2eq), 1.97 (1H, m, H2ax). Furthermore, the ^{1}H NMR spectrum disclosed the presence of two pairs of aromatic ABX protons at δ_H 7.08 (1H, d, J = 2.0 Hz, H2' and H2''), 6.98 (1H, brd, J = 8.3 Hz, H6' and H6''), 6.80 (1H, d, J = 8.1 Hz, H-5' and H-5''), and four trans-oriented olefinic protons at δ_H 7.63, (each 1H, d, J = 16.5 Hz, H7' and H-7'') and 6.30 (each 1H, d, J = 16.5 Hz, H-8', H-8''), which were attributed to a trans-caffeoyl moiety. The existence of two caffeoyl units was clear based on the integration of the proton peaks. The large coupling constant of 16.5 Hz between the olefinic protons of the caffeoyl moiety suggested a trans-configuration.

The ^{1}H–^{1}H COSY spectrum showed coupling between the oxygenated methines H3, H4, and H5 with proton resonances at ca.2 ppm for H2 and H6, which suggested the presence of a quinic acid nucleus that is responsible for the isolated spin system highlighted in green

lines and box (Figure S5). The coupling protons for the ABX (yellow line and box) and olefinic (blue line and box) systems were observed in the downfield region between 6.0 and 8.0 ppm.

The quaternary carbons were detected using HMBC correlation (Figure S6), there were three carbonyls (C9’, C9’‘, and C7 at 167.2, 167.2, and 176.5 ppm, respectively); four hydroxylated aromatic carbons C3’, C4’, C3’‘, C4’‘ at 147.5, 146.5, 147.5 and 146.5 ppm, respectively; an alkyl-substituted aromatic carbon for C1’ and C1’‘ at 121.6 ppm; and one oxygenated aliphatic quaternary carbon at 71.7 ppm for C1.

There were no clear HMBC correlations that supported the connectivity of two caffeoyl residues on the quinic acid group. The caffeoyl moiety had an ester linkage with a hydroxyl group of the quinic acid unit, which deshielded the corresponding methine proton significantly downfield. Alternately, if signals of the quinic acid ring protons H3, H4, and H5 were shifted upfield, then the caffeoyl group would be attached to the hydroxyl group at C1 of the quinic acid as earlier described in the literature [1]. The proton signals of H-3 (4.94 ppm) and H-5 (5.86) were significantly deshielded, implying that the attachment of C-1 of the caffeoyl moiety to the hydroxyl group of the quinic acid unit. Accordingly, full assignment of the spectral data of F8-ChJ was identified as 3,5-O-dicaffeoylquinic acid [2]. The ^{1}H and ^{13}C NMR assignments are presented in Table S1.

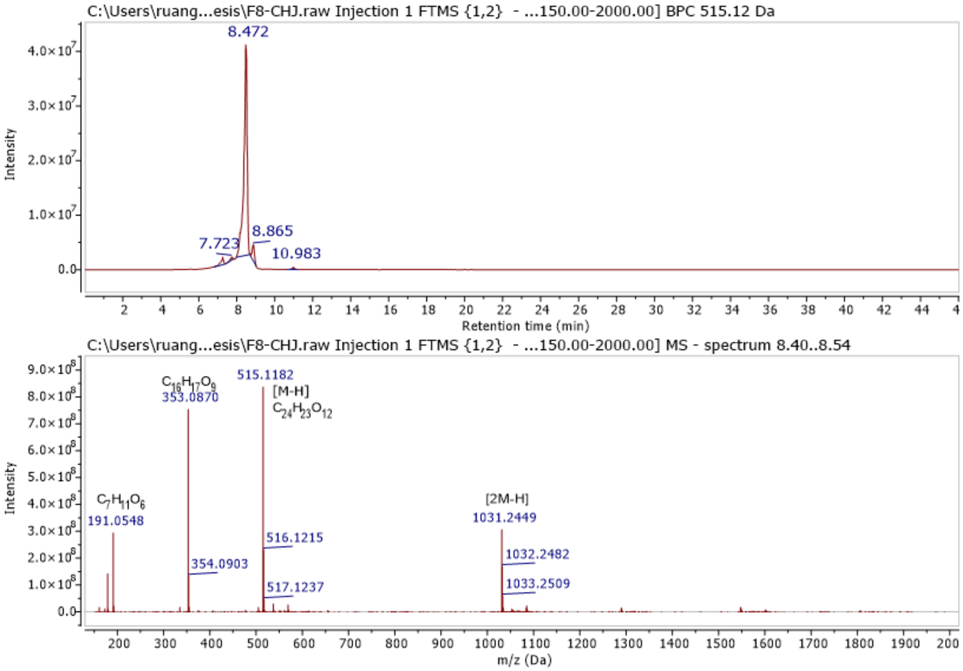

Figure S2. Extracted ion chromatogram and mass spectrum of F8-ChJ from the Jordanian chamomile showing source fragmentation at m/z 515.1182 [M−H]− eluting at 8.47 min

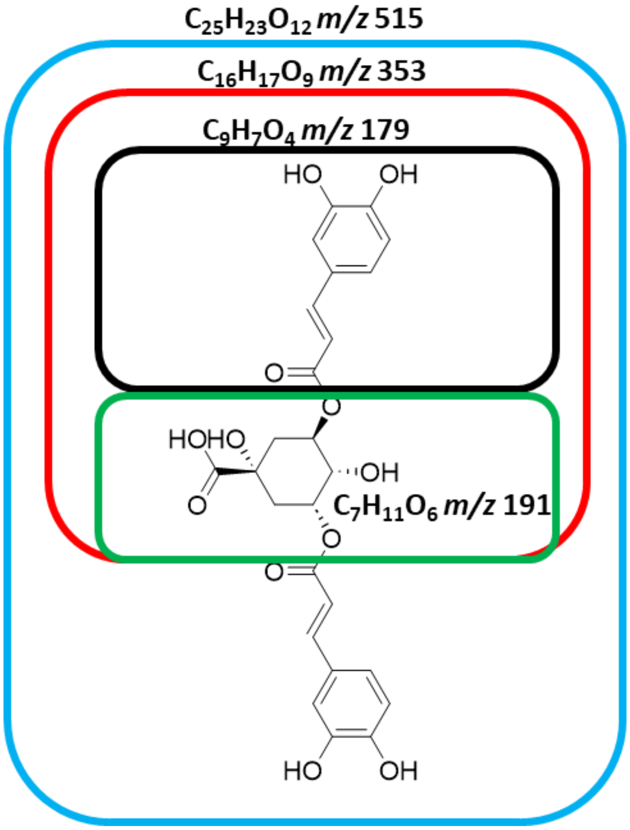

Figure S3. Fragmentation scheme of a dicaffeoylquinic acid isomer.

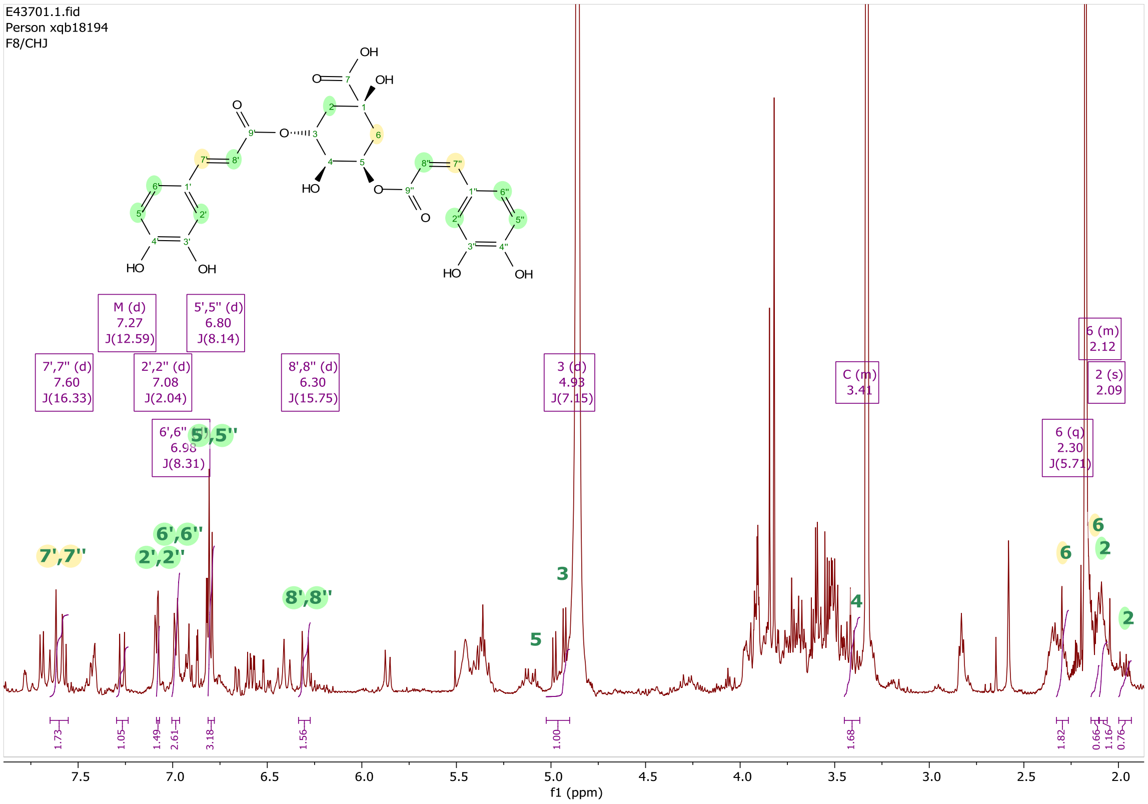

Figure S4: ^{1}H NMR spectrum of F8-ChJ in CD_3OD measured at 400 MHz.

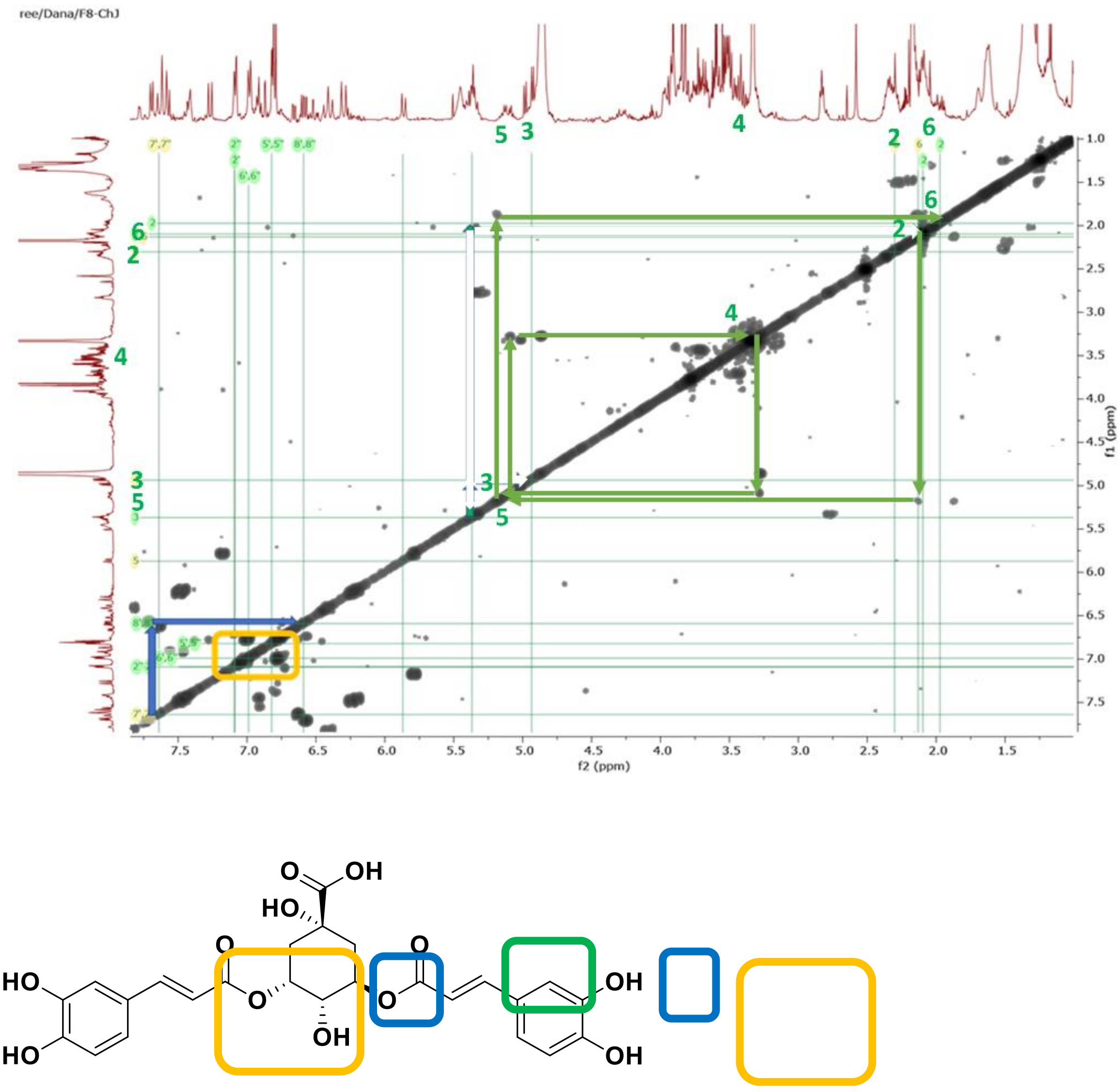

Figure S5: (^{1}H-^{1}H) COSY correlation spectrum of F8-ChJ in CD_3OD measured at 400 MHz.

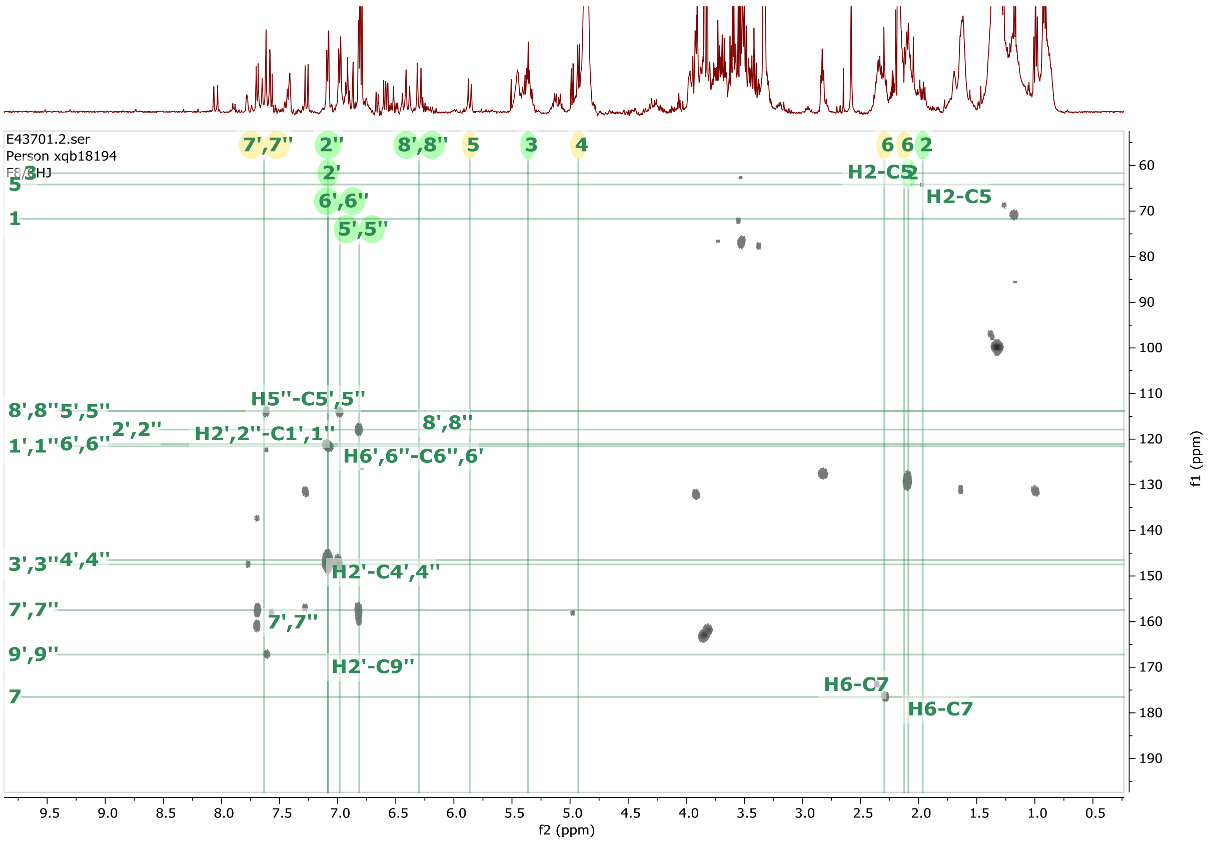

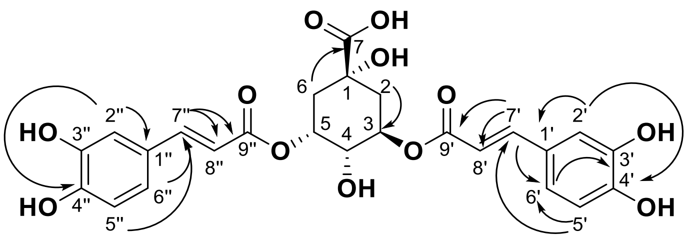

Figure S6: HMBC spectrum of F8-ChJ in CD_3OD measured at 400 MHz.

Table S1. ^{1}H and ^{13}C NMR data of F8-ChJ in CD_3OD.

**Position**	**δ_C**	**δ_H (mult, *J* in Hz)**	**COSY**	**HMBC**
**1**	71.7 qC			
**2**	38.0 CH_2	2.09, 1.97	3	
**3**	61.7 CH	5.36 t (6)	2, 4	
**4**	64.1 CH	4.94 dd (13.0, 6.0)	3, 5	
**5**	64.2 CH	5.86 d (13.1)	4, 6	
**6**	39.7 CH_2	2.30 q (5.7)<br>2.12 m	5	C7
**7**	176.5 qC			
**1′**	121.6 qC			
**2′**	117.9 CH	7.08 d (2.0)		C1′, C4′
**3′**	147.5 qC			
**4′**	146.5 qC			
**5′**	113.9	6.80 d (8.1)	6′	C7′
**6′**	121.0 CH	6.98 brd, (8.3)	5′	C3′, C5′
**7′**	157.4 CH	7.60 d, (16.3)	8′	C5′, C6′, C8′, C9′
**8′**	113.7 CH	6.30 d, (15.7)	7′	
**9′**	167.2 qC			
**1′′**	121.6 qC			
**2′′**	117.9 CH	7.08 d (2.0)		C1′′, C4′′
**3′′**	147.5 qC			
**4′′**	146.5 qC			
**5′′**	113.9 CH	6.80 d (8.1)	6′′	C7′′
**6′′**	121.0 CH	6.98 brd, (8.3)	5′′	C3′′, C5′′
**7′′**	157.4 CH	7.60 d, (16.3)	8′′	C5′′, C6′′, C8′′, C9′′
**8′′**	113.7 CH	6.30 d, (15.7)	7′′	
**9′′**	167.2 qC			

2 Structural elucidation of biological active fractions in European chamomile.

2.1 Structure elucidation of F2-ChE from European chamomile.

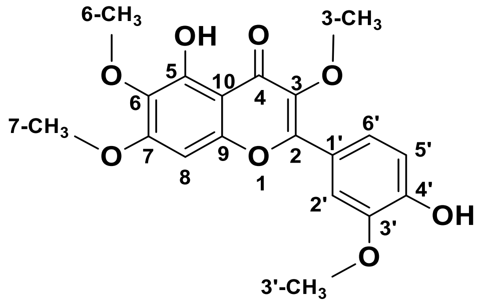

Figure S7. Chemical structure of F2-ChE, chrysosplenetin, Chemical Formula: $C_{19}H_{18}O_8$, MW: 374.1000

F2-ChE (Figure S7) afforded a high yield with a TIC peak area of 3.64E+09. HRMS established a molecular formula of $C_{19}H_{19}O_8$ with an ion peak at *m/z* 375.1080 $[M+H]^+$ and 749.2094 $[2M+H]^+$ eluting at 15.5 min (Figure S8). In the negative mode, the ion peak at *m/z* 373.0927 $[M-H]^-$ was observed for the molecular formula $C_{19}H_{17}O_8$ (Figure S9). Fraction F2-ChE was 3% of a gram of the crude extract affording 30 mg of chrysosplenetin at 80% purity as indicated by the ^{1}H nmr shown in Figure S10.

The ^{1}H NMR spectrum of F2-ChE in Figure showed the presence of four methoxy S10 singlets at δ_H 3.98 (3H, s, H7), 3.88 (3H, s, H5'), 3.82 (3H, s, H3), and 3.74 (3H, s, H6), and four aromatic protons at δH 7.69 (1H, d, *J*=2.07 Hz, H6'), 7.64 (1H, dd, *J*=2.07 and 8.45 Hz, H2'), 6.97 (1H, d, J=8.45 Hz, H3'), 6.94 (1H, s, H8). The multiplicities and coupling constants of H2', H3', and H6' were typical of a tri-substituted aromatic ABX system. In addition, two hydroxyl signals at δ_H 12.64 (s, 5-O*H*) and 9.97 (s, 4'-O*H*) were observed.

The HMBC spectrum shown in Figure S11 helped assigned the ^{13}C resonances for F2-ChE, which included δ_C 159.3 (C2), 137.9 (C3), 178.6 (C4), 152.2 (C5), 131.9 (C6), 162.7 (C-7), 155.6 (C9), 105.9(C10), 121.0 (C1′), 112.2 (C3′), 150.6 (C4′), 155.5 (C5′), and 121.1 (C6′). HMBC correlations disclosed the presence of four aromatic methine carbons (through cross peaks with C3' and C6' at 112.3, and 121.1 ppm, respectively), and ten non-protonated carbons (four of them were aromatic (C2, C9, C10, and C1' at 159.3, 155.6, 105.9, and 121.0 ppm, respectively), two quaternary aromatic attached to a hydroxyl group (C4' and C5 at 150.6, and 152.2 ppm, respectively), four quaternary

aromatic carbons attached to methoxy groups (C3, C6, C7, C3' at 137.9, 131.9, 162.7, and 155.5, respectively) and one carbonyl ketone at 178.6 ppm for C4.

HMBC correlations in Figure S11 established the connectivity of the substructures postulated from the ^{1}H NMR spectral data. Correlations from 5-O*H* to C5, C6 and C10 ascertained ring A of a flavonoid structure. Allocating ring C was achieved from correlations between H2''to C3', C4' and C5' as well as H5' to C6'. Furthermore, the HMBC cross peak between H8 at 4.98 ppm with C5, C6, C7, and 10 was used to identify ring B. The structure of F2-ChE was identified as 4',5-dihydroxy-3,3',6,7-tetramethoxyflavone also known as chrysosplenetin. NMR spectral data of F2-ChE presented in Table S2 was compatible and identical to chrysosplenetin as described in the literature [3].

Formula	Calculated Mass	Target Mass	Double Bond Equivalence	Absolute Error (ppm)	Error (mDa)	Error (ppm)	Fitness
$C_{19}H_{18}O_8$	375.10744	375.10803	11.0	1.56	0.58	1.56	1.000

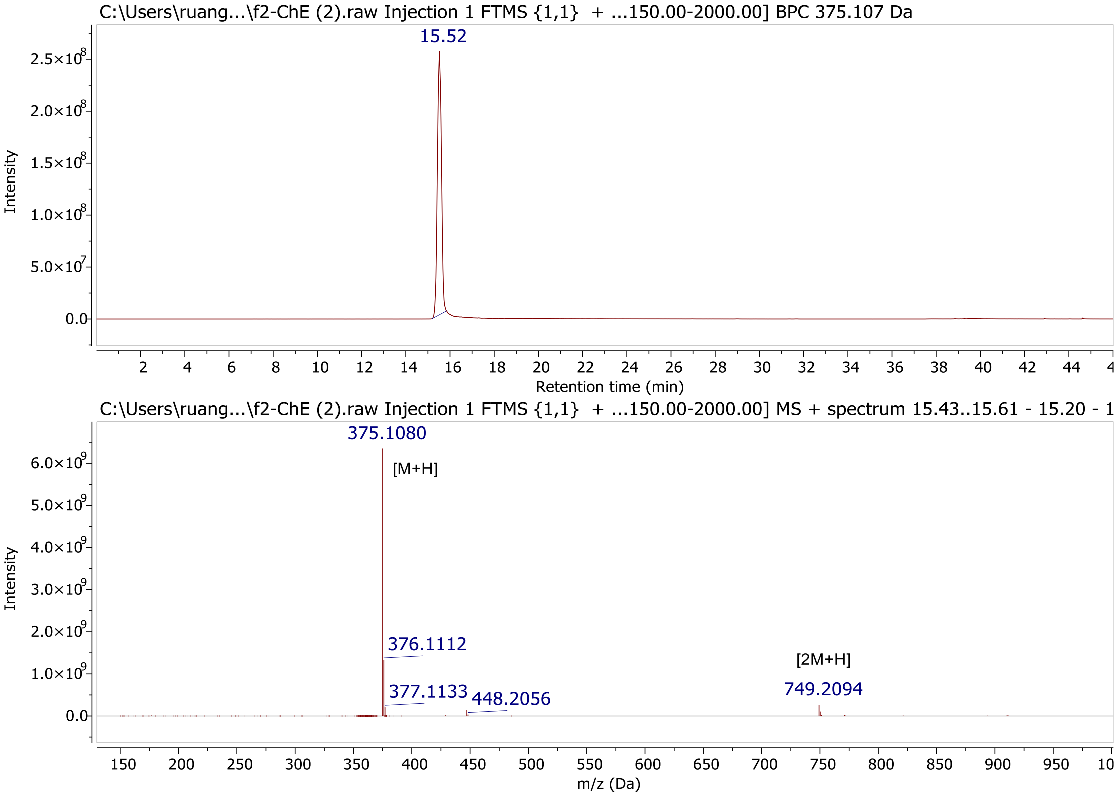

Figure S8. Extracted ion chromatogram of F2-ChE for the ion peak at *m/z* 375.1080 $[M+H]^{+}$at a RT of 15.5 min.

Formula	Calculated Mass	Target Mass	Double Bond Equivalence	Absolute Error (ppm)	Error (mDa)	Error (ppm)	Fitness
$C_{19}H_{17}O_8$	373.09234	373.09271	11.5	0.98	0.36	0.98	1.000

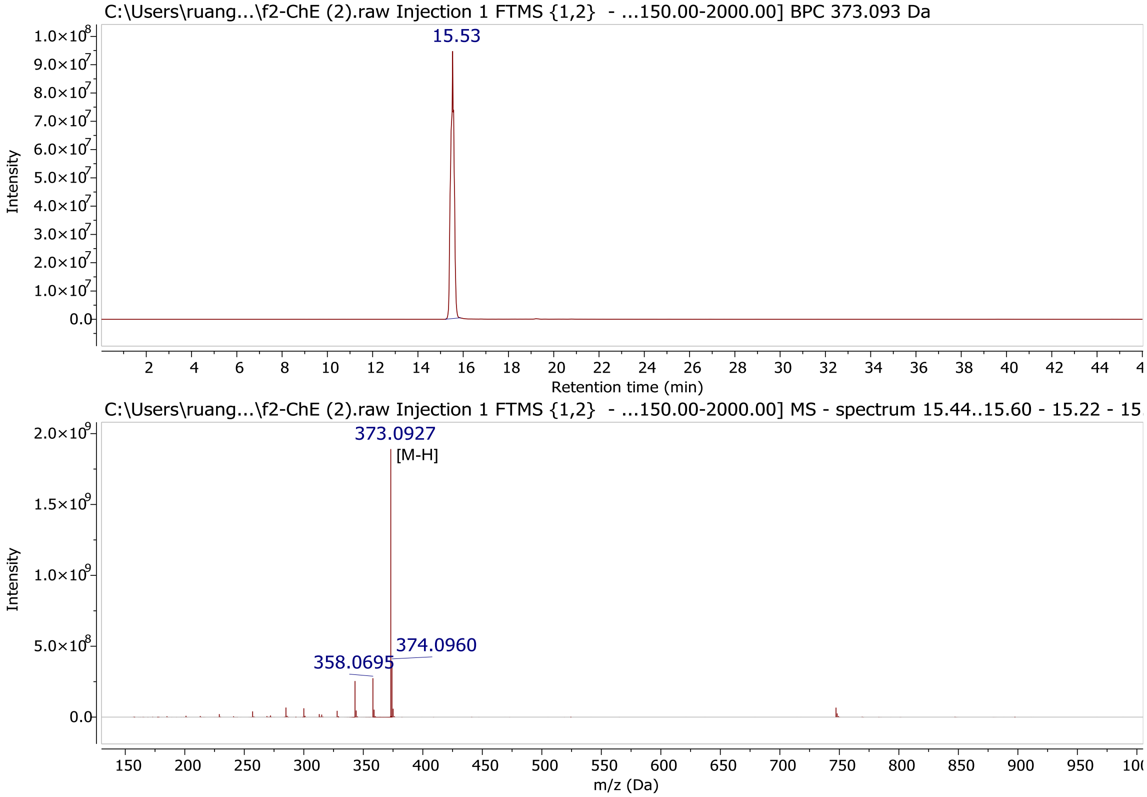

Figure S9. Extracted ion chromatogram of F2-ChE for the ion peak at *m/z* 373.0927 [M-H]$^-$ at a RT of 15.5 min.

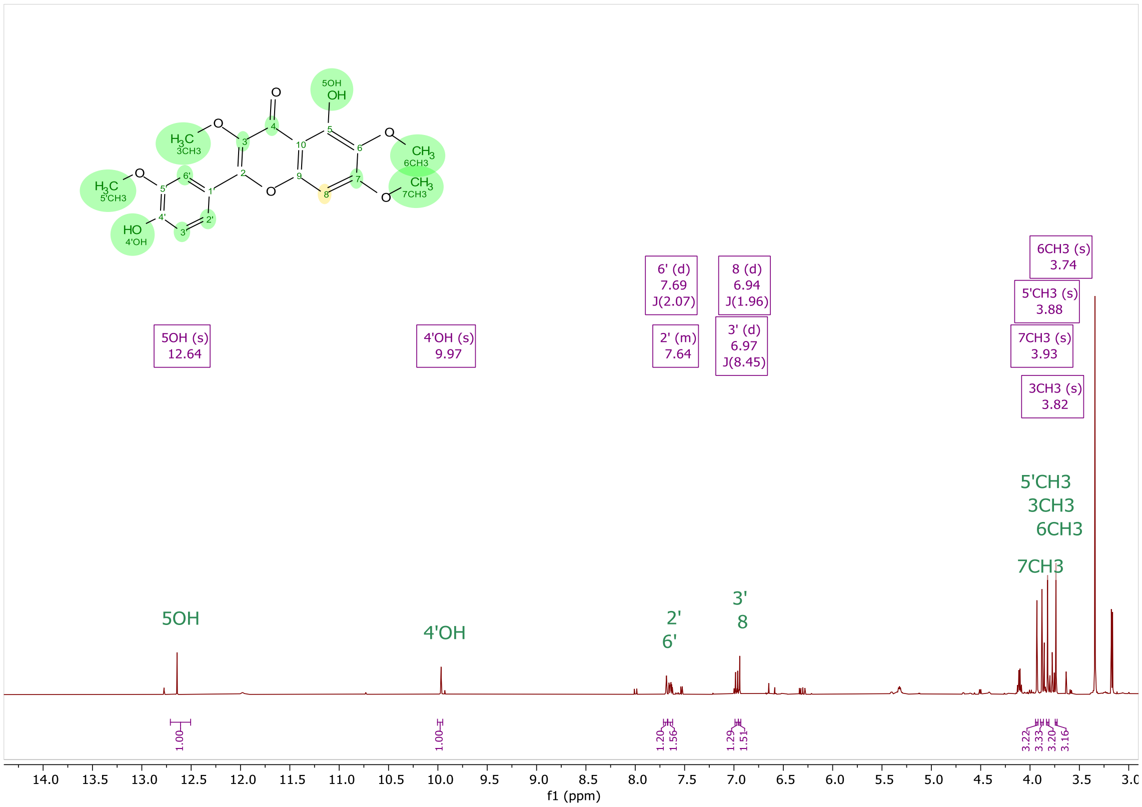

Figure S10: ^{1}H NMR spectrum of F2-ChE in DMSO-d_6 measured at 400 MHz.

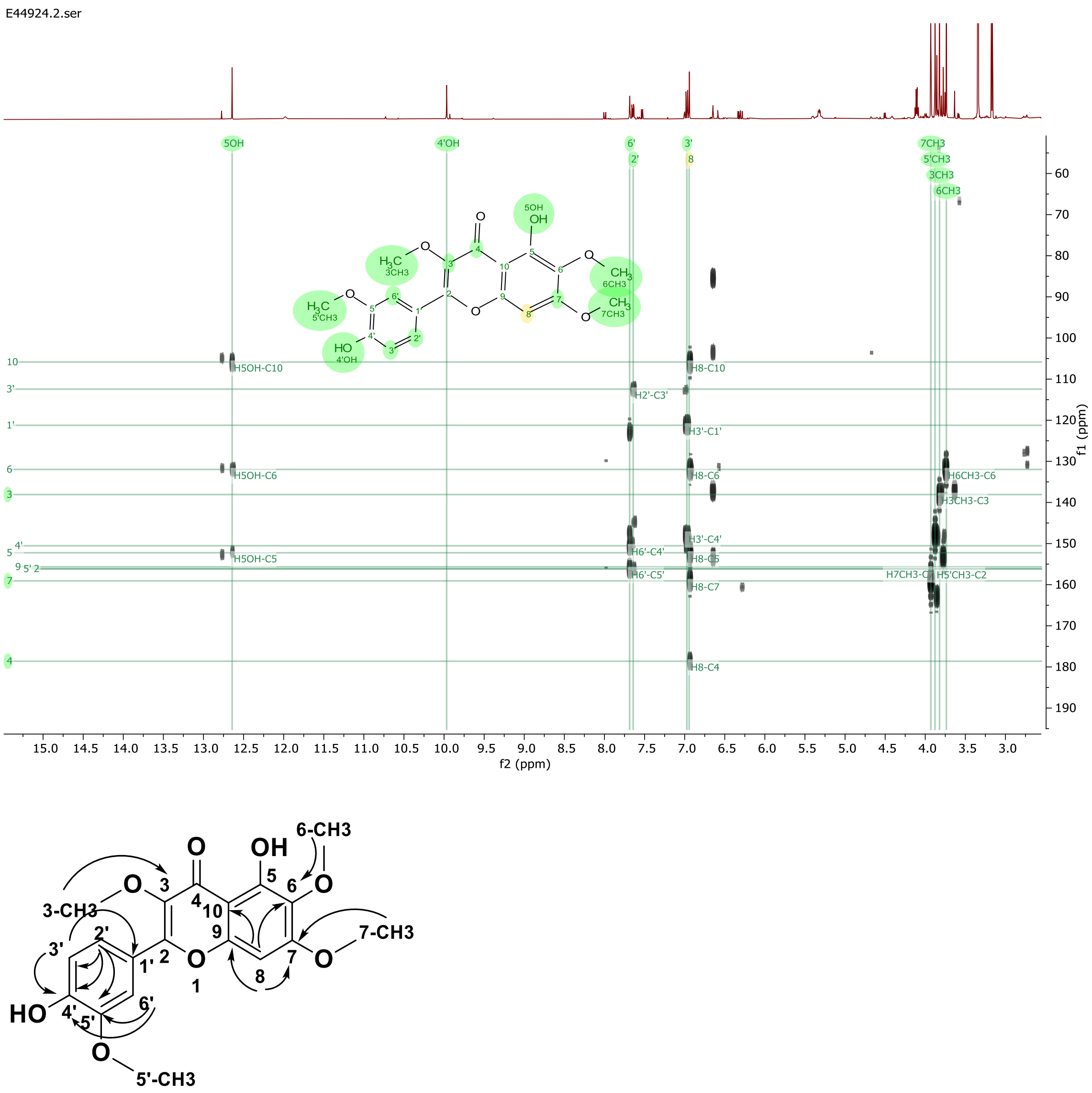

Figure S11: HMBC spectrum of F2-ChE in DMSO-d_6 measured at 500 MHz.

Table S2: ^{1}H and ^{13}C NMR data of F2-ChE in comparison to chrysosplenetin [3]

**Atom no.**	**^{13}C (mult) in MeOH-d_6***	**^{13}C lit. in $CDCl_3$**	**^{1}H lit. (mult) in DMSO-d_6 *J* in Hz**		**^{1}H (mult) in DMSO-d_6 *J* in Hz**		**HMBC**
**2**	159.3 qC	156.10					
**3**	137.9 qC	138.73					
**4**	178.6 qC	178.95					
**5**	152.2 qC	152.79					
**6**	131.9 qC	132.34					
**7**	162.7 qC	158.86					
**8**	n.d.	90.45	6.94 s		6.94 s		C5, C6, C7, C9, C10
**9**	155.6 qC	152.38					
**10**	105.9 qC	106.61					
**1′**	121.0 qC	122.45					
**2′**	n.d.	110.99	7.65 dd	2.14, 8.33	7.64 dd	2.0, 8.5	C3′, C4′, C5′
**3′**	112.2 CH	114.72	6.97 d	8.39	6.97 d	8.5	C1′, C4′
**4′**	150.6 qC	148.51					
**5′**	155.5 qC	146.51					
**6′**	121.1 CH	122.68	7.69 d	2.14	7.69 d	2.0	C4′, C5′
**5-OH**	n.d.		12.64 s		12.64 s		C5, C6, C10
**4′-OH**	n.d.		9.97 s		9.97 s		
**6-OCH_3**	n.d.	60.98	3.74 s		3.74 s		C6
**3-OCH_3**	n.d.	60.25	3.82 s		3.82 s		C3
**3′-OCH_3**	n.d.	56.22	3.88 s		3.88 s		C5′
**7-OCH_3**	n.d.	56.44	3.93 s		3.98 s		C7
***^{13}C assignment obtained from HMBC spectrum; n.d. (not detected)**							

2.2 Structure elucidation of F8-ChE from European chamomile.

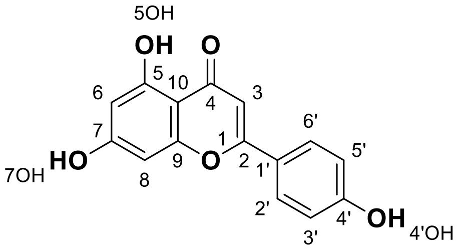

Figure S12. Chemical structure of F8-ChE, apigenin, Chemical Formula: $C_{15}H_{10}O_5$, Exact Mass: 270.0525

F8-ChE gave an ion peak at *m/z* 269.0474 for $C_{15}H_9O_5$ $[M-H]^-$ and 539.1022 $[2M-H]^-$ at a RT of 12.5 min and a peak area of 1.015E+09 (Figure S13). In the positive mode, an ion peak was observed at *m/z* 271.0600 $[M+]^+$ (Figure S14). F8-ChE gave a yield of 8 mg. The structure of F8-ChE was shown in Figure S12. Fraction F8-ChE was 0.8% of a gram of the crude extract affording 8 mg of apigenin at 60% purity as indicated by the TIC in Figure 8 and ^{1}H nmr shown in Figure S15.

The ^{1}H NMR spectrum in Figure S15 revealed the presence of three hydroxyl singlets at δ_H 12.96 (5-O*H*), 10.34 (4'-O*H*) and 10.19 (7-O*H*); seven aromatic protons at δ_H 7.93 (2H, dd, *J* = 9.4, 2.9 Hz, H2′, H6′), 6.93 (2H, m, H3′, H5′), 6.78 (1H, s, H-3), 6.48 (1H, d, *J* = 2.1 Hz, H8), and 6.19 (^{1}H, d, *J* = 2.1 Hz, H6). The resonances at δ_H 7.93 and 6.93 that integrated for 2H each with a coupling constant of 9.4 Hz for a doublet indicated an AA'BB' system. Dereplication of the mass spectral data of the bioactive fractions putatively identified the presence of 4',5,7-trihydroxyisoflavone or apigenin. Comparison of the extrapolated NMR resonances observed for F8-ChE to those of a synthesized apigenin [4], both measured in DMSO-d6 at 400 MHz, afforded similar spectral data (Table S3). The structure of F8-ChE was elucidated then elucidated as apigenin.

Formula	Calculated Mass	Target Mass	Double Bond Equivalence	Absolute Error (ppm)	Error (mDa)	Error (ppm)	Fitness
$C_{15}H_9O_5$	269.04555	269.04744	11.5	7.04	1.89	7.04	1.000

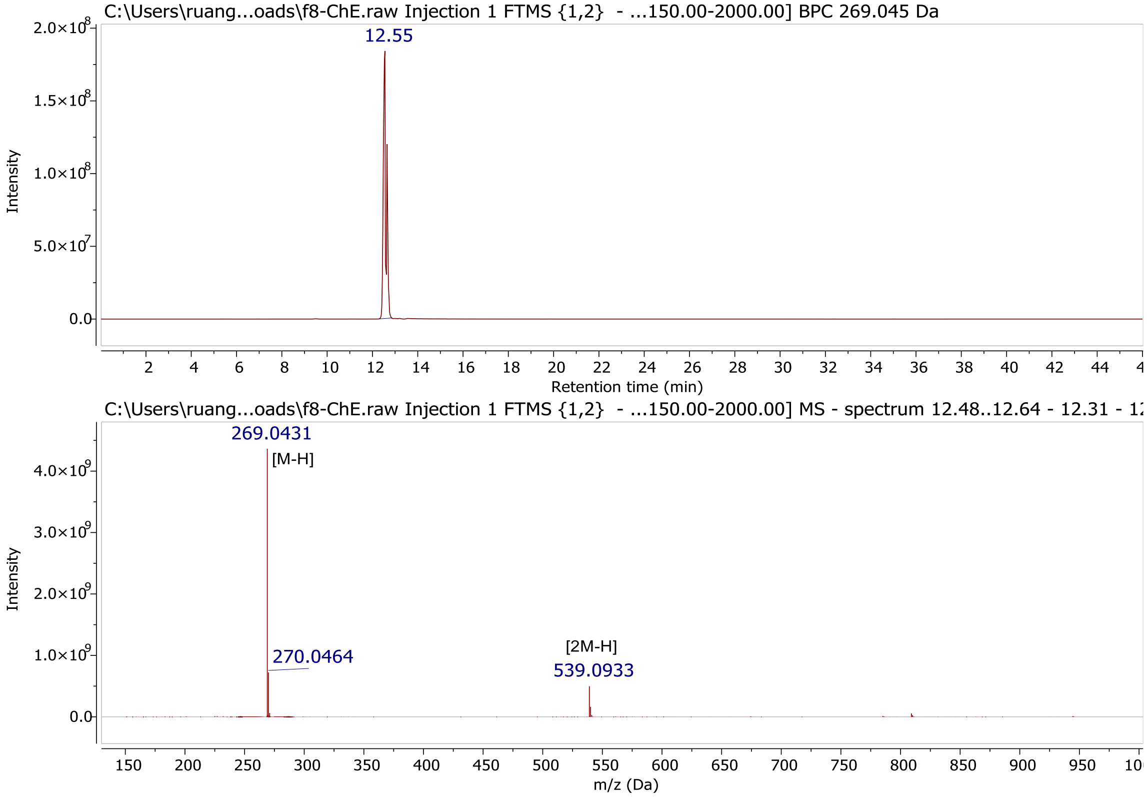

Figure S13. Extracted ion chromatogram of F8-ChE for the ion peak at *m/z* 269.0450 $[M{-}H]^-$ eluting at RT of 12.5min.

Formula	Calculated Mass	Target Mass	Double Bond Equivalence	Absolute Error (ppm)	Error (mDa)	Error (ppm)	Fitness
$C_{15} H_{10} O_5$	271.06010	271.06000	11.0	0.38	-0.10	-0.38	1.000

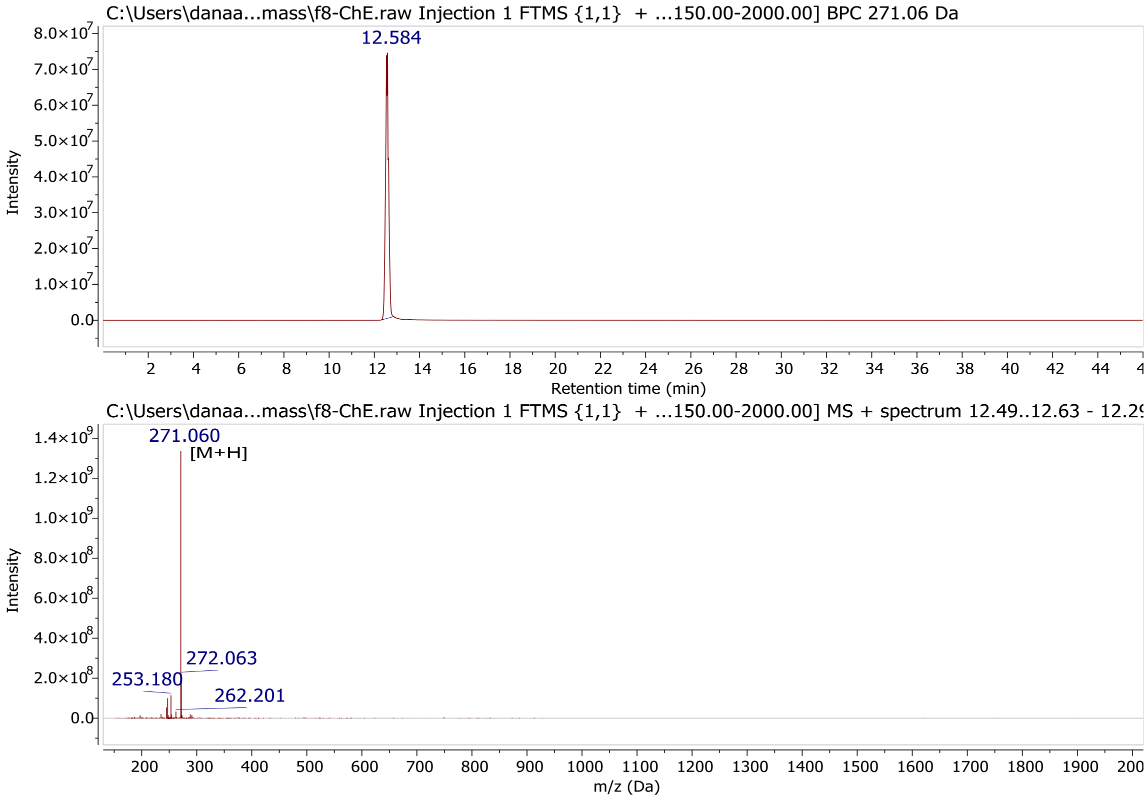

Figure S14. Extracted ion chromatogram of F8-ChE for the ion peak at *m/z* 271.0600 $[M+H]^+$ eluting at RT of 12.5 min.

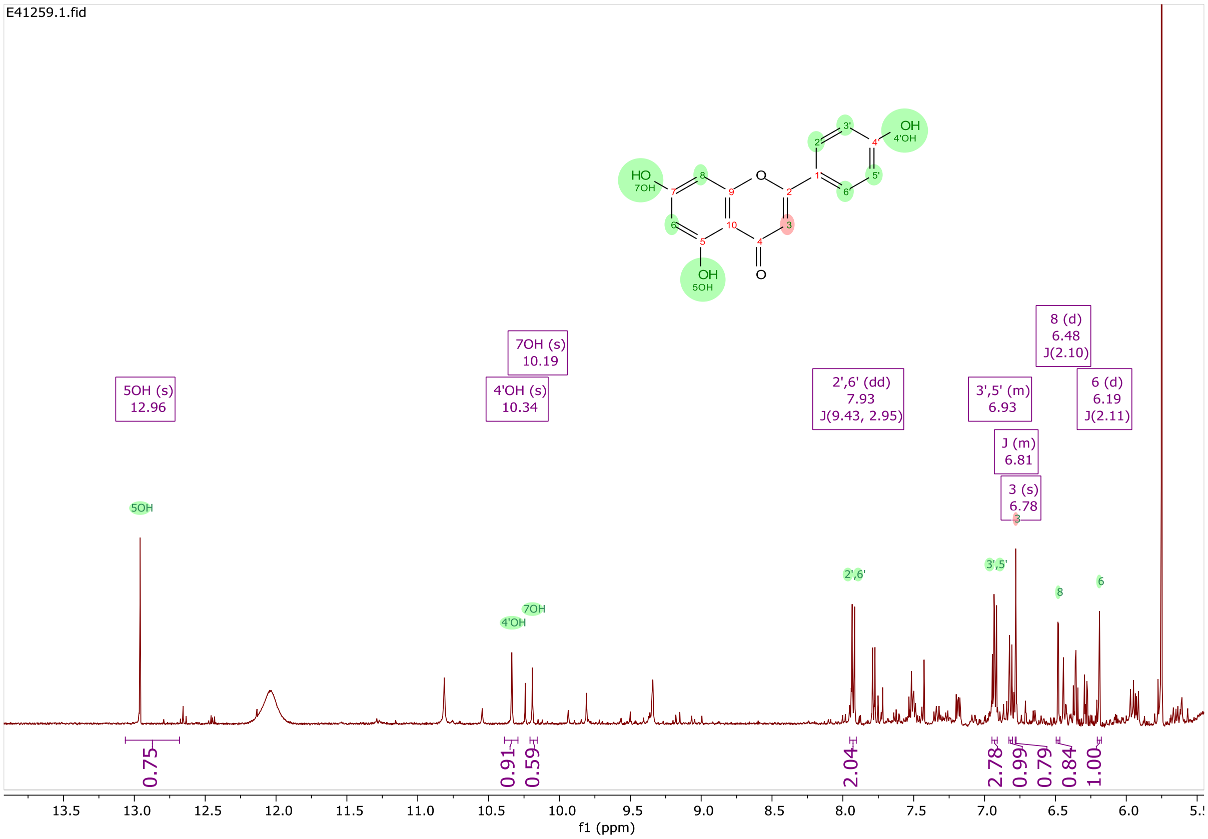

Figure S15: ^{1}H NMR spectrum of F8-ChE in DMSO-d_6 measured at 400 MHz.

Table S3: ^{1}H NMR data of F8-ChE and synthesised apigenin measured at 400 MHz.

Atom no.	^{1}H (mult) F8-ChE (DMSO-d_6)	^{1}H (mult) synthesised apigenin (DMSO-d_6)
**3**	6.78 s	6.75 s
**6**	6.19 d, J = 2.1 Hz	6.17 d, J = 2.11 Hz
**8**	6.48 d, J = 2.1 Hz	6.46 d, J = 2.10 Hz
**2′**	7.93 dd, J = 9.4, 2.9 Hz	7.91 d, J = 9.43 Hz
**3′**	6.93 m	6.94 m
**5′**	6.93 m	6.94 m
**6′**	7.93 dd, J = 9.4, 2.9 Hz	7.91 d, J = 9.43 Hz
**5-OH**	12.96 s	12.95 s
**4′-OH**	10.34 s	10.50 overlapping brs
**7-OH**	10.19 s	10.50 overlapping brs

2.3 Structure elucidation of F11-ChE from European chamomile.

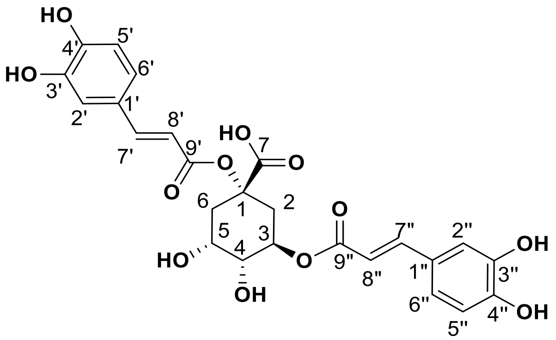

1,3-*O*-dicaffeoylquinic acid

Figure S16. Chemical structure of F11-ChE, Chemical Formula: $C_{25}H_{24}O_{12}$, MW: 516.1261

F11-ChE (Figure S16) was isolated as a yellow amorphous powder with a yield of 10.8 mg. Mass spectrometric results suggested a DBE of 14, which accounted for three rings, eight double bonds from the aromatic system, and three carbonyl groups as part of the structure. HREIMS established a chemical formula of $C_{25}H_{24}O_{12}$ for the ion peak at *m/z* 517.1338 $[M+H]^+$, in addition to ion peaks at *m/z* 1033.2612 $[2M+H]^+$ and 1549.3885 $[3M+H]^+$ Figure S17. In the negative mode, an ion peak was observed at *m/z* 515.1179 $[M-H]^-$ and 1031.2451 $[2M-H]^-$ at a RT of 8.1 min (Figure S18). Fraction F11-ChE was 1.1% of a gram of the crude extract affording 11 mg of 1,3-*O*-dicaffeoylquinic acid at 50% purity as indicated by the TIC in Figure 8 and ^{1}H nmr shown in Figure S19.

^{1}H NMR spectrum of F11-ChE (Figure S19) was comparable to that of F8-ChJ and are structural isomers having the same MWs and gave a difference of 0.3 seconds in their elution time. Although the spectral data of F11-ChE and F8-ChJ, they were still not identical. Both respective isomers were found to be the most abundant in each of the varieties. However, the spectral data of both metabolites were very different to that of any of di-acetyl cosmosiin analogues, which are acylated apigenin glycosides reported from *M. chamomilla* , [5]. Although apigenin was a major component in the European variety as implied by the TIC in Figure 9, the bioactivity of the extracts was led by the di-O-caffeoylquinic acid analogues found in fractions F11-ChE and F8-ChJ.

The ^{1}H NMR spectral data of F11-ChE displayed the typical trans olefinic doublet pairs of a propenoic acid moiety or caffeoyl unit at approximately 6.5 and 7.5 ppm as well as the characteristic coupling pattern for 1,3,4-trisubstituted phenyl ABX system. However, in comparison to the ^{1}H NMR spectrum of F8-ChJ**,** there was a more distinct separation of the trans olefinic ^{1}H signals for the two caffeoyl units. Instead of observing twice the

number of integrations for two overlapping protons like in F8-ChJ, resonances for the trans olefinic doublet pairs and meta doublets gave two sets of chemicals shifts for each of the caffeoyl moieties as presented in Table S4. The chemical shift of the methine proton on C3 was deshielded downfield in F11-ChE, which indicated the esterification of the caffeoyl unit on 3-OH and loss of caffeoyl moiety on C2, C4, C5 because these carbons were shielded upfield. The second caffeoyl unit was attached on the hydroxyl unit on C1 when compared to the literature (Table S4).

The ^{1}H assignments for F11-ChE and esterification on C3 were also established by a COSY spectrum as shown in Figure S21. Commencing upfield at 1.94 and 2.14 ppm for H2A and H2B, a cross peak was observed downfield at 5.20 ppm for H3, which continued to 3.40 ppm for H4 then to 3.27 ppm for H5 and finally ended upfield at 1.69 and 1.84 ppm for H6A and H6B. By inspection and comparison of the spectral data of F11-ChE (Table S4) with those reported in the literature, the isolated compound was elucidated as 1,3-*O*-dicaffeoylquinic acid [2,6-8]. However, in the literature there has been a confusion between the nomenclatures of 1,5- and 1,3-*O*-dicaffeoylquinic acid. A classic example is cynarin, isolated from artichokes, was first reported as 1,3-di-*O*-caffeoyl-D-(−)-quinic acid [7] but were being drawn as the 1,5 analogue on databases and monographs (e.g. Dictionary of Natural Products, PubMed). Confirmation of the structure for a 1,3 analogue would entail isopropylidene derivatisation of the free vicinal hydroxyl units in cis configuration to form an O-ketal ring [9]. The quinic acid moiety bears three vicinal hydroxyl groups where C4 and C5 are *cis*-oriented while C3 and C4 are trans to each other. Hence, a selective acetylation would only occur for 1,3-O-dicaffeoylquinic acid to form the 4,5-isopropylidene ketal ring Figure S20.

Formula	Calculated Mass	Target Mass	Double Bond Equivalence	Absolute Error (ppm)	Error (mDa)	Error (ppm)
$C_{25}H_{25}O_{12}$	517.13460	517.13383	13.5	1.50	-0.77	-1.50

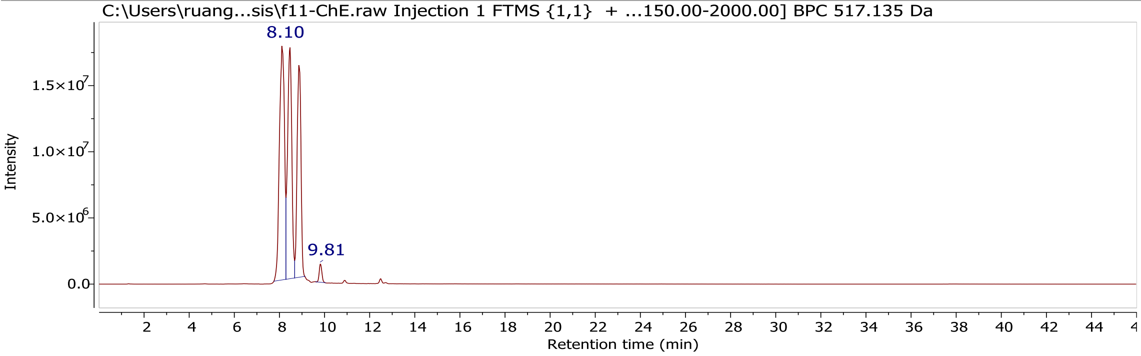

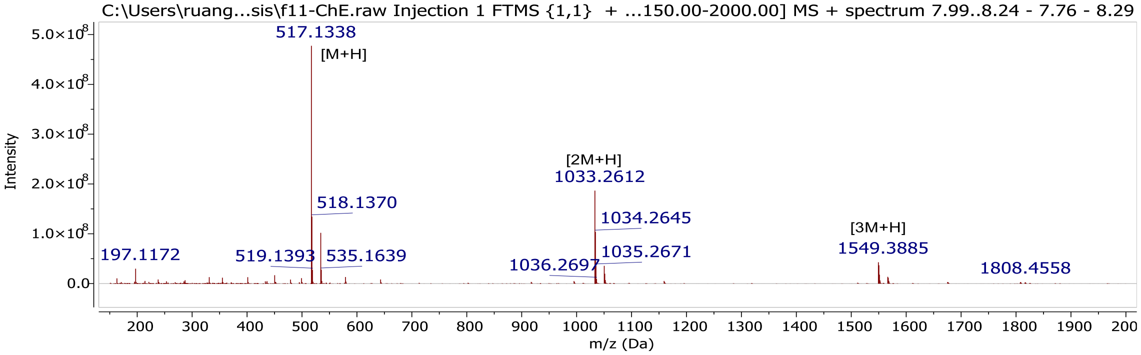

Figure S17. Extracted ion chromatogram of F11-ChE for the ion peak at *m/z* 517.1338 $[M+H]^+$ with a RT of 8.1 min.

Formula	Calculated Mass	Target Mass	Double Bond Equivalence	Absolute Error (ppm)	Error (mDa)	Error (ppm)
$C_{25}H_{23}O_{12}$	515.11895	515.11753	14.5	2.76	-1.42	-2.76

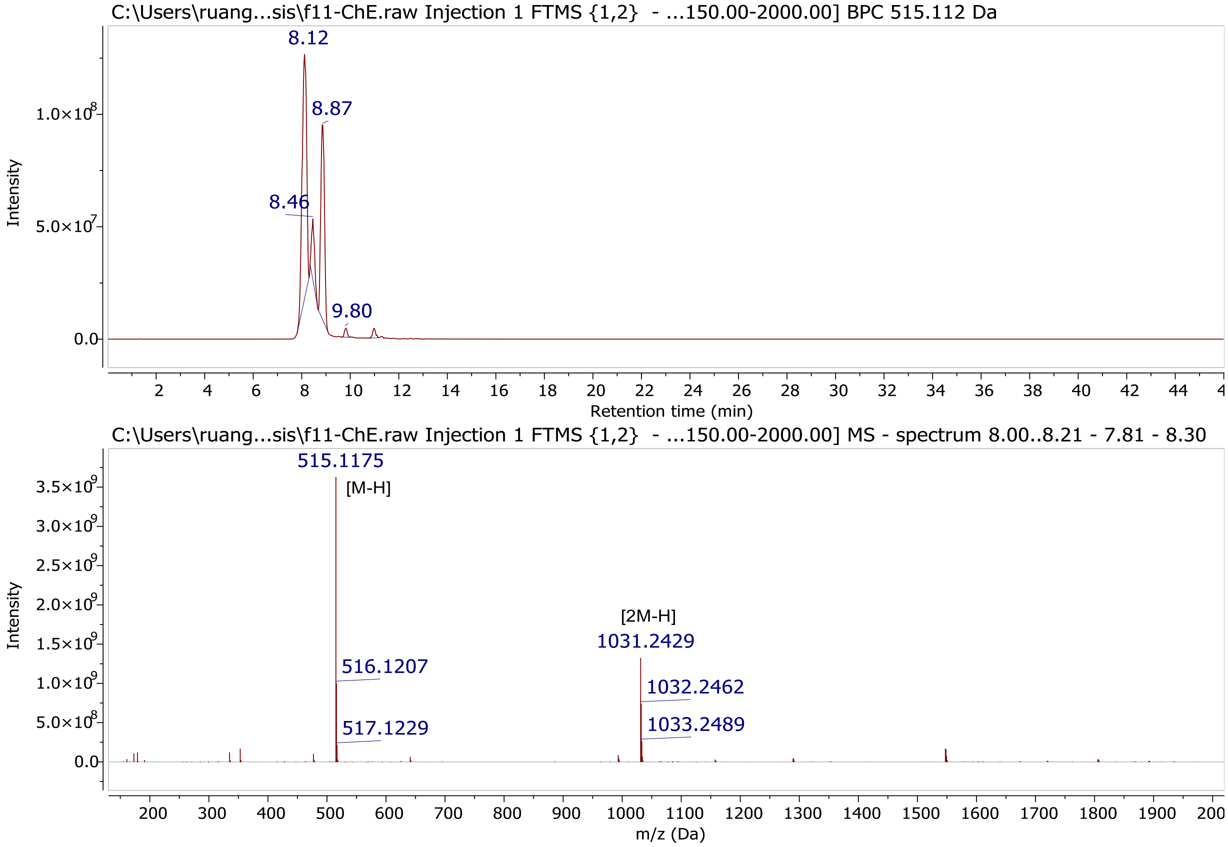

Figure S**18.** Extracted ion chromatogram of F11-ChE for the ion peak at *m/z* 515.1117 $[M{-}H]^-$ with a RT of 8.1 min.

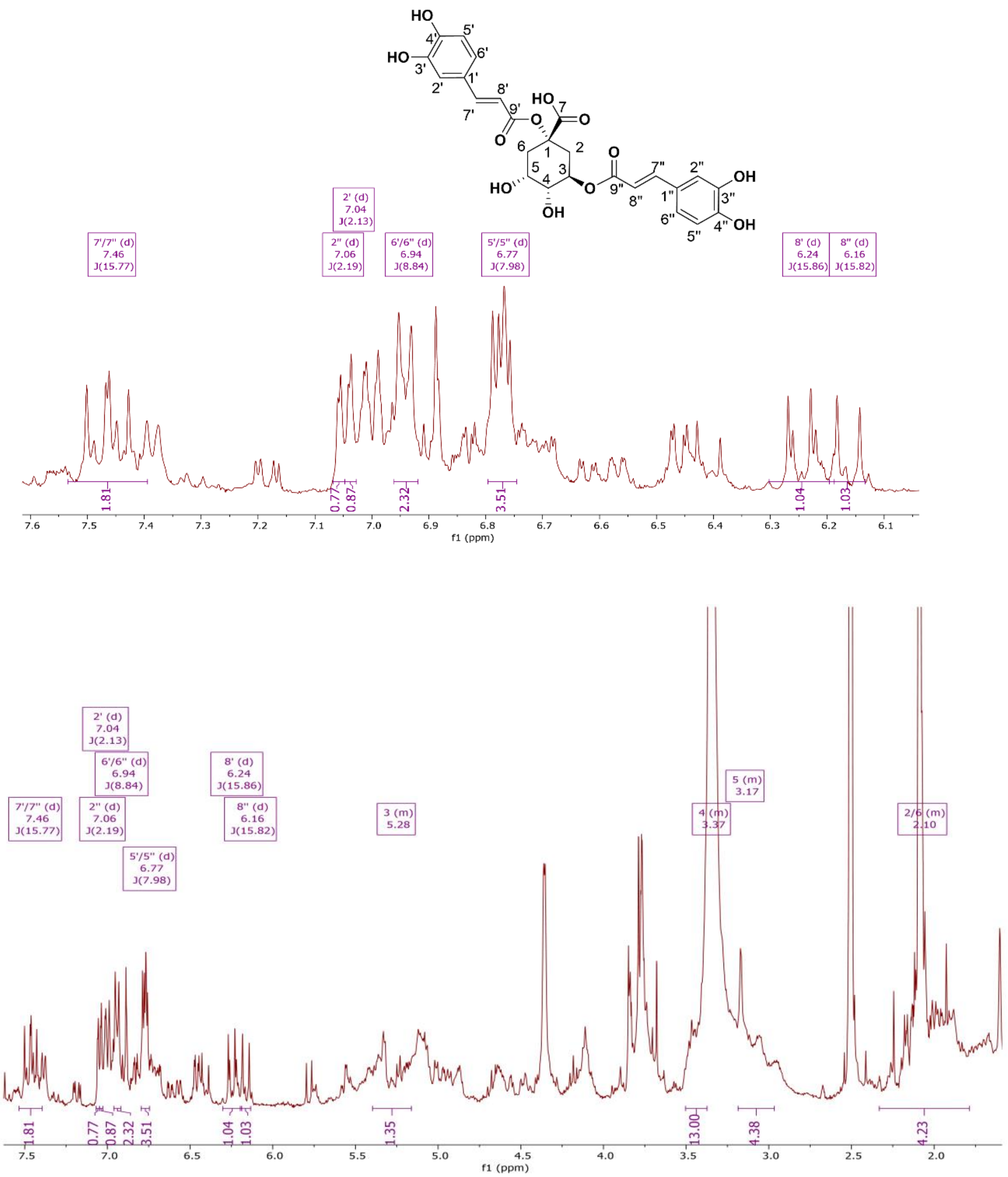

Figure S19: ^{1}H NMR spectrum of F11-ChE in DMSO-d_6 measured at 400 MHz.

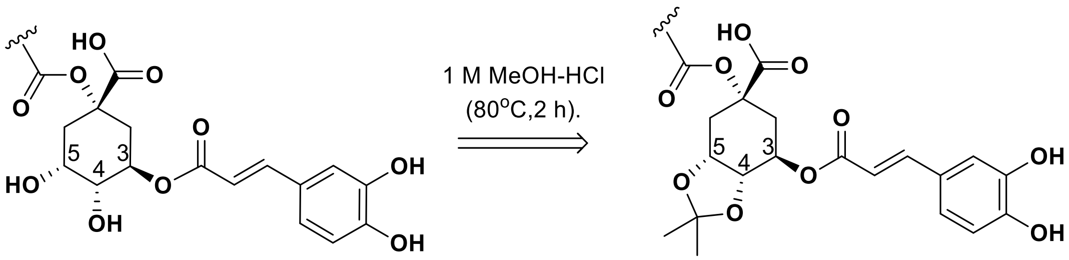

Figure S20. Isopropylidene derivatisation of 1,3-*O*-dicaffeoylquinic acid

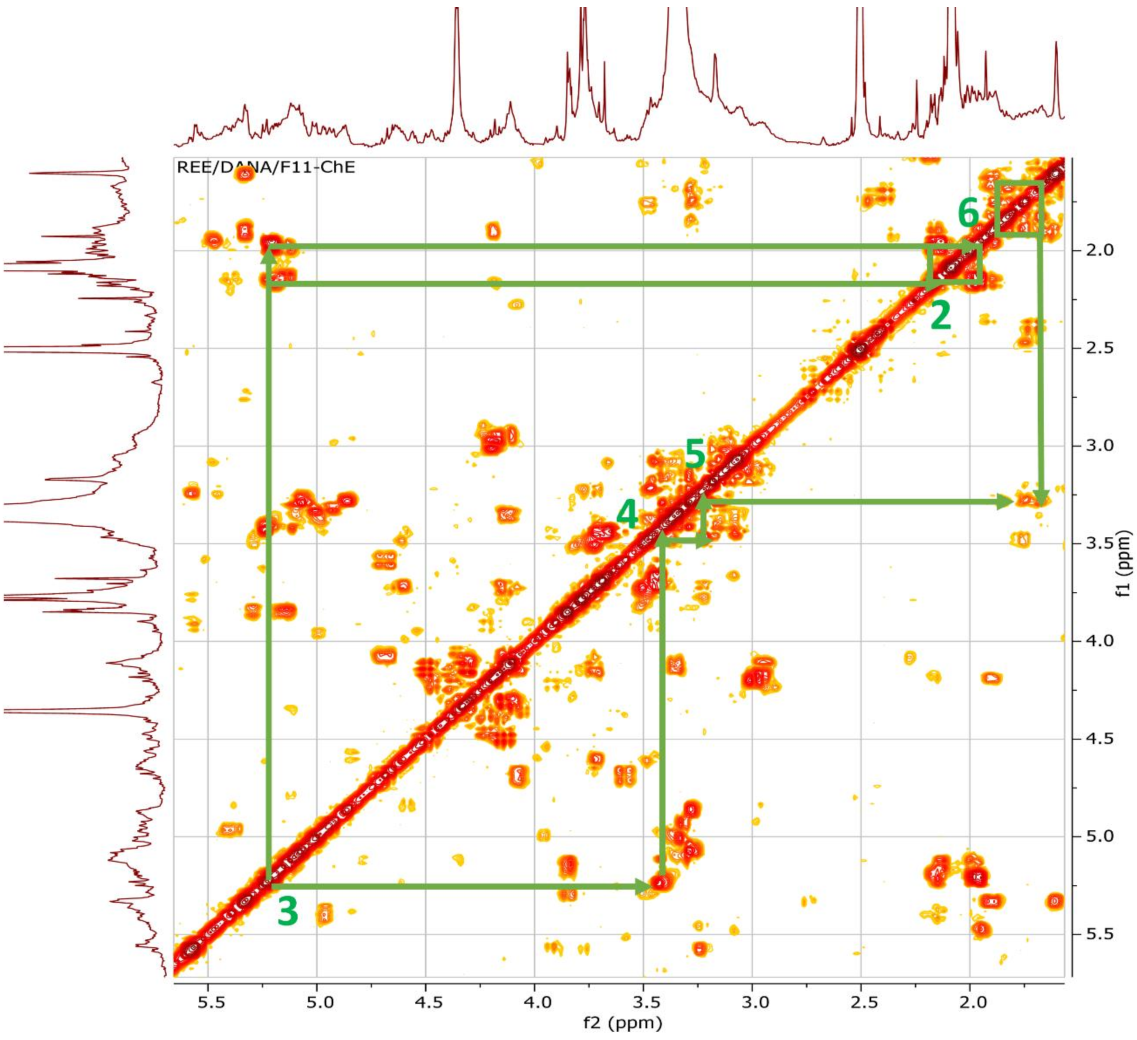

Figure S21. COSY NMR spectrum of the quinic acid moiety in F11-ChE measured in DMSO-d_6 at 400 MHz.

Table S4. ^{1}H NMR data(δ_H in ppm, mult, *J* in Hz) of F11-ChE in comparison to cynarin, a 1,3-dicaffeoylquinic acid analogue along with spectral data of 1,5-dicaffeoylquinic acid reported in the literature.

**Atom no.**	**^{1}H (mult, *J* in Hz) F11-ChE (DMSO-d_6) 400 MHz**	**1,3-dicaffeoylquinic acid* [8] (D_2O) 400 MHz**	**1,5-dicaffeoylquinic acid [10] (MeOD) 400 MHz**	**1,5-dicaffeoylquinic acid [11] (D_2O) 500 MHz**
2	1.94 m<br><br>2.14 m	2.29 axial<br>(2.34 dd, *J* = 3.5, 15.5)§<br>2.88 equatorial<br>(2.77 ddd, *J* = 3.5, 3.0, 15.5)§	2.43 m<br><br>2.54 dd, *J* = 3.5, 10.2	2.22 dd, *J* = 3.5, 15.5<br><br>2.50 ddd, *J* = 3.8, 2.8, 15.5
3	5.20 m	5.36 equatorial<br>(5.43 ddd, *J* = 3.5. 3.5, 3.5)§	4.28 brd, *J* = 3.5	4.27 ddd, *J* = 3.5, 3.8, 3.5
4	3.40 m	4.23 axial<br>(4.67 dd, *J* = 9.5, 3.5)§	3.76 dd, *J* = 3.0, 8.1	3.86 dd, *J* = 9.6, 3.5
5	3.27 m	3.62 axial<br>(4.23 ddd, *J* = 9.5, 4.5, 10.5)§	5.37 ddd, *J* = 3.7, 8.1, 8.1	5.29 ddd, *J* = 9.6, 4.3, 10.8
6	1.69 m<br><br>1.84 m	1.83 axial<br>(1.88 dd, *J* = 10.5, 13.5)§<br>2.53 equatorial<br>(2.50 ddd, *J* = 4.5, 3.0, 13.5)§	2.05 dd, *J* = 11.1, 13.8<br><br>2.43 m	1.99 dd, *J* = 10.8, 13.8<br><br>2.56 ddd, *J* = 4.3, 2.8, 13.8
2′	7.04 d, *J* = 2.1	6.81	7.04 brs	7.12 d, *J* = 2.1
5′	6.77 d, *J* = 8.0	6.88 d, *J* = 8.0 Hz	6.78 d, *J* = 8.1	6.88 d, *J* = 8.2
6′	6.94 br d, *J* = 8.8	6.58	6.96 d, *J* = 8.1	7.05 dd, *J* = 8.2, 2.1
7′	7.48 d, *J* =15.9	7.58 d, *J* = 16.0 Hz	7.58 d, *J* =15.9	7.59 d, *J* = 16.1
8′	6.25 d, *J* =15.9	6.32 d, *J* = 16.0 Hz	6.27 d, *J* =15.9	6.39 d, *J* = 16.1
2″	7.06 d, *J* = 2.2	6.81	7.04 brs	7.15 d, *J* = 2.1
5″	6.77, d, *J* = 8.0	6.88 d, *J* = 8.0 Hz	6.76 d, *J* = 8.1	6.86 d, *J* = 8.2
6″	6.94 br d, *J* = 8.8	6.58	6.94 d, *J* = 8.1	7.08 dd, *J* = 8.2, 2.1
7″	7.45 d, *J* =15.8	7.58 d, *J* = 16.0	7.55 d, *J* =15.9	7.58 d, *J* = 16.2
8″	6.16 d, *J* =15.8	6.32 d, *J* = 16.0	6.24 d, *J* =15.9	6.32 d, *J* = 16.2

*Identified as cynarin by the authors. §Spectral data set reported by Horman *et al.*, (1984) for quinic acid of cynarin in acetone-d_6 measured at 300MHz.

2.4 Structure elucidation of F12-ChE from European chamomile.

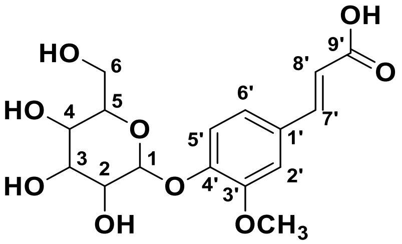

Figure S22. Chemical structure of F12-ChE, 4'→1-*O*-feruloylglucose, chemical Formula: $C_{16}H_{20}O_9$ MW: 356.1179

F12-ChE was obtained as a white amorphous powder with a yield of 2.0 mg. High resolution mass spectrometric data gave a MW of 356.1179 Da and established a chemical formula of C16H20O9 for the ion peak at *m/z* 355.1030 [M-H]-, in addition to ion peaks at *m/z* 711.2130 [2M-H]- and 1067.3230 $[3M-H]^-$ Figure S23. In the positive mode, an ion peak was observed at *m/z* 357.1180 $[M+H]^+$ in addition to 713.2291 [2M+H]+, 1069.3402 [3M+H]+, and 1425.4507 [4M+H]+ Figure S24. Source fragmentation of the ion peak at *m/z* 357.1180 yielded the peak at *m/z* 195.065 that corresponded to the aglycone structure upon the loss of a glucose unit Figure S25. F12-ChE was presented in Figure S22. Fraction F12-ChE was 0.2% of a gram of the crude extract affording 2 mg of 4'→1-*O*-feruloylglucose at 60% purity as indicated by the ^{1}H nmr shown in Figure S26.

The ^{1}H NMR spectrum of F12-ChE in Figure S26 was almost identical to that of F11-ChE as it is a mixture of the components found in the latter fraction at almost 1:1 ratio that was demonstrated by an almost equal integrals of the olefinic protons found in both structures. F12-ChE and F11-ChE share the component, 1,3-*O*-dicaffeoylquinic acid, that is the main metabolite from the latter fraction, which is the major impurity in F12-ChE and vice versa. Due to the very low yields of both fractions, it was not feasible to subject them to further purification work. Both fractions, F11-ChE and F12-ChE, exhibited antioxidant activities with EC_{50} values of 0.311 (603 µM) and 0.165 (463 µM) mg/mL, respectively. F12-ChE displayed a higher potency than F11-ChE, which could be due to the additional effectiveness of the component in F12-ChE or could also be a result of synergistic biological activity of the two components.

Furthermore, the ^{1}H NMR spectrum of F12-ChE disclosed a comparable pattern of resonating proton signals for a trans olefinic doublet pair of a propenoic acid moiety at 7.83 and 6.41 ppm with a coupling constant of 16.3 Hz, like those found for the structures of 1,3-O-dicaffeoylquinic acid (F11-ChE) and 3,5-O-dicaffeoylquinic acid (F8-ChJ). The

characteristic coupling pattern for 1,3,4-trisubstituted phenyl system was also observed in the ^{1}H NMR spectrum of F12-ChE. However, in comparison to the ^{1}H NMR spectra (Table S5) of the dicaffeoyl analogues elucidated in F8-ChJ and F11-ChE, the ortho-coupled doublet on C5' went downfield by 1 ppm at δ_H 7.96 (J = 8 Hz) and the meta-coupled doublet on C2' was shifted upfield by 0.3 ppm at δ_H 6.73 (J = 3 Hz), while the ortho-meta doublet of doublet on C6' remained unchanged at δ_H 6.95 (J = 3.1, 7.9 Hz). These changes in chemical shifts of the ABX system in the aromatic region implied a modification in substituents on the phenyl ring. The presence of a methoxy signal that was not apparent in the ^{1}H NMR spectrum of F8-ChJ for 3,5-*O*-dicaffeoylquinic acid, indicated a ferulic acid unit in F12-ChE with the methylation of the hydroxyl group in C3'observed at 3.78 ppm. Nevertheless, the largest ^{1}H shift change affected the proton doublet on C5', which postulated the possible glycosylation on C4'.

The ^{1}H assignments for the feruloyl moiety and the glucose unit were established by correlation NMR spectroscopy (COSY) as shown Figure S27. On the aromatic region is the feruloyl unit, which is represented by the blue line for the propenoic acid moiety, and the yellow line is the ABX system of the 1,3,4-trisubstituted phenyl system of the ferulic acid unit that commenced at 7.83 and 7.96 ppm, respectively. The green line did reveal the presence of a glucose unit that started at the anomeric ^{1}H doublet at 5.01 ppm with a coupling constant of 7.1 Hz, which suggested a β-D-glucose configuration [12]. The ^{1}H assignments for the glucose substituent followed through δ_H 3.35 (1H, m, H2), 3.19 (1H, m, H3), 3.01 (1H, m, H4), 3.12 (1H, m, H5), and concluded at 3.46 (2H, m, H6).

In Table S5, F12-ChE was also compared to 1-*O*-feruloyl-β-D-glucose [13], where the chemical shifts of the feruloyl units were more comparable to those of dicaffeoylquinic acid analogues found in F8-ChJ and F11-ChE. In 1-*O*-feruloyl-β-D-glucose, the glucose unit is attached to the propenoic acid unit like in the caffeoylquinic acid congeners. Further comparison to literature data is shown in Table S6. The structure of F12-ChE was elucidated as 4'→1-*O*-feruloylglucose, also known as *Z*-glucoferulic acid [14] or lavandoside [15].

Formula	Calculated Mass	Target Mass	Double Bond Equivalence	Absolute Error (ppm)	Error (mDa)	Error (ppm)
$C_{16}H_{20}O_9$	357.11801	357.11799	7.0	0.07	-0.02	-0.07

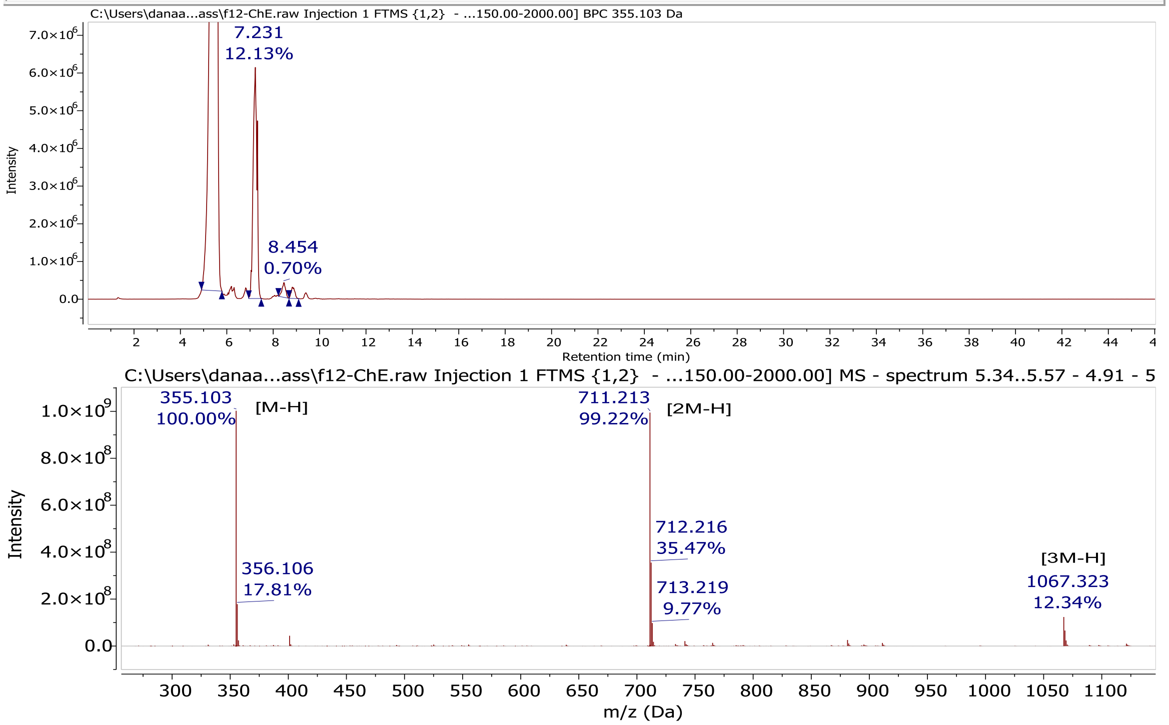

Figure S23. Extracted ion chromatogram of F12-ChE for the ion peak at *m/z* 355.1030 [M-H] eluting at a Rt of 7.23 min.

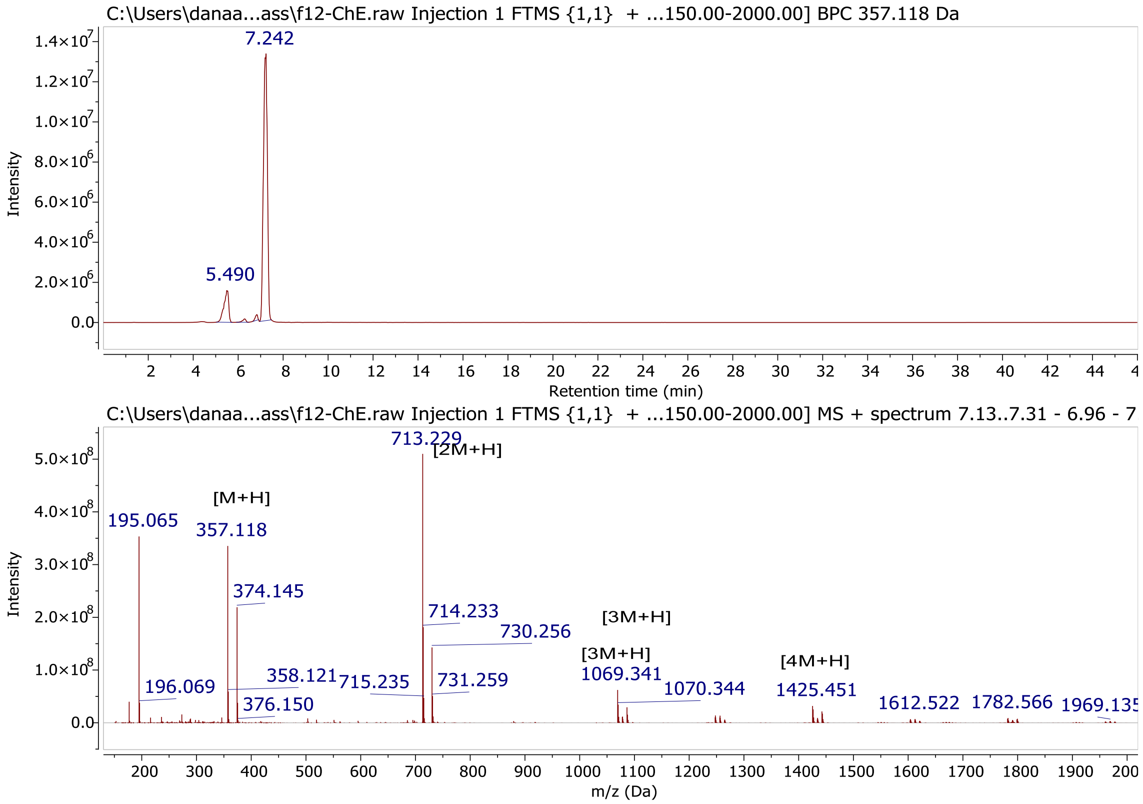

Figure S24. Extracted ion chromatogram of F12-ChE for the ion peak at *m/z* 357.1180[M+H] eluting at a Rt of 7.24 min.

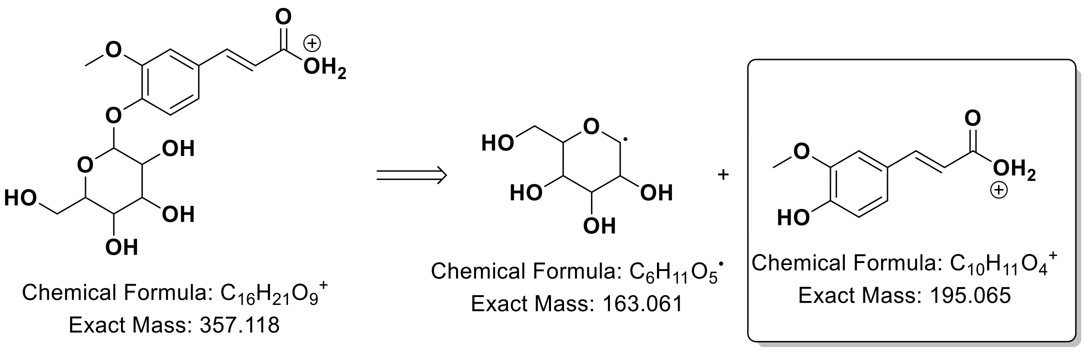

Figure S25. Source fragmentation of the ion peak at *m/z* 357.1180 of F12-ChE.

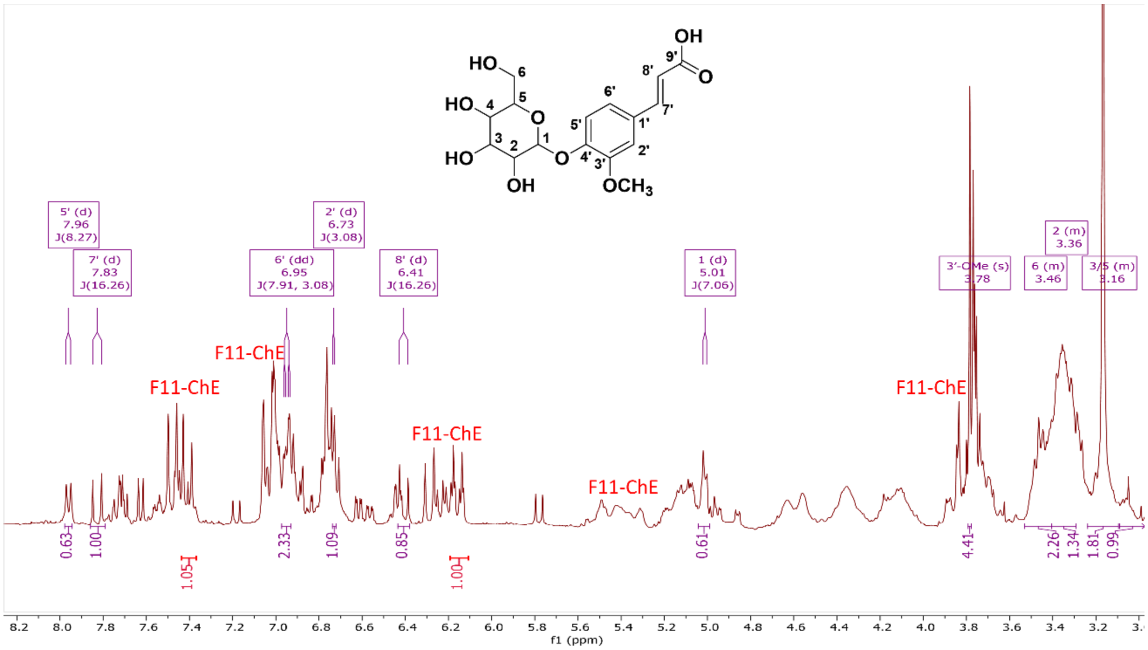

Figure S26: ^{1}H NMR spectrum of F12-ChE in DMSO-d_6 measured at 400 MHz.

Table S5: ^{1}H NMR data (δ_H in ppm, mult, *J* in Hz) of F12-ChE in comparison to that of 1,3-*O*-dicaffeoylquinic acid (F11-ChE) and 3,5-*O*-dicaffeoylquinic acid (F8-ChJ) measured at 400 MHz in DMSO-d_6.

**Atom no.**	**4’→1-*O*-feruloylglucose F12-ChE**	**1,3-*O*-dicaffeoylquinic acid F11-ChE**	**3,5-*O*-dicaffeoylquinic acid F8-ChJ**
1	5.01 d, *J* = 7.1		
2	3.41 – 3.29 m	2.14 m<br>1.94 m	2.09<br>1.97
3	3.24 – 3.10 m	5.20 m	4.96 m
4	3.09 – 2.93 m	3.40 m	3.41 m
5	3.24 – 3.10 m	3.27 m	5.11 m
6	3.53 – 3.40 m	1.69 m<br>1.84 m	2.30 q, *J* = 5.7<br>2.12 m
2'	6.73 d, *J* = 3.1	7.04 d, *J* = 2.1<br>7.06 d, *J* = 2.2	7.08 d, *J* = 2.0<br>7.08 d, *J* = 2.0
5'	7.96 d, *J* = 8.3	6.77 d, *J* = 8.0<br>6.77, d, *J* = 8.0	6.80 d, *J* = 8.1<br>6.80 d, *J* = 8.1
6'	6.95 dd, J = 3.1, 7.9	6.94 br d, *J* = 8.8<br>6.94 br d, *J* = 8.8	6.98 brd, *J* = 8.3<br>6.98 brd, *J* = 8.3
7'	7.83 d, *J* = 16.3	7.48 d, *J* =15.9<br>7.45 d, *J* =15.8	7.60 d, *J* = 16.3<br>7.60 d, *J* = 16.3
8'	6.41 d, *J* = 16.3	6.25 d, *J* =15.9<br>6.16 d, *J* =15.8	6.30 d, *J* = 15.7<br>6.30 d, *J* = 15.7
3'-OCH_3	3.78 s		

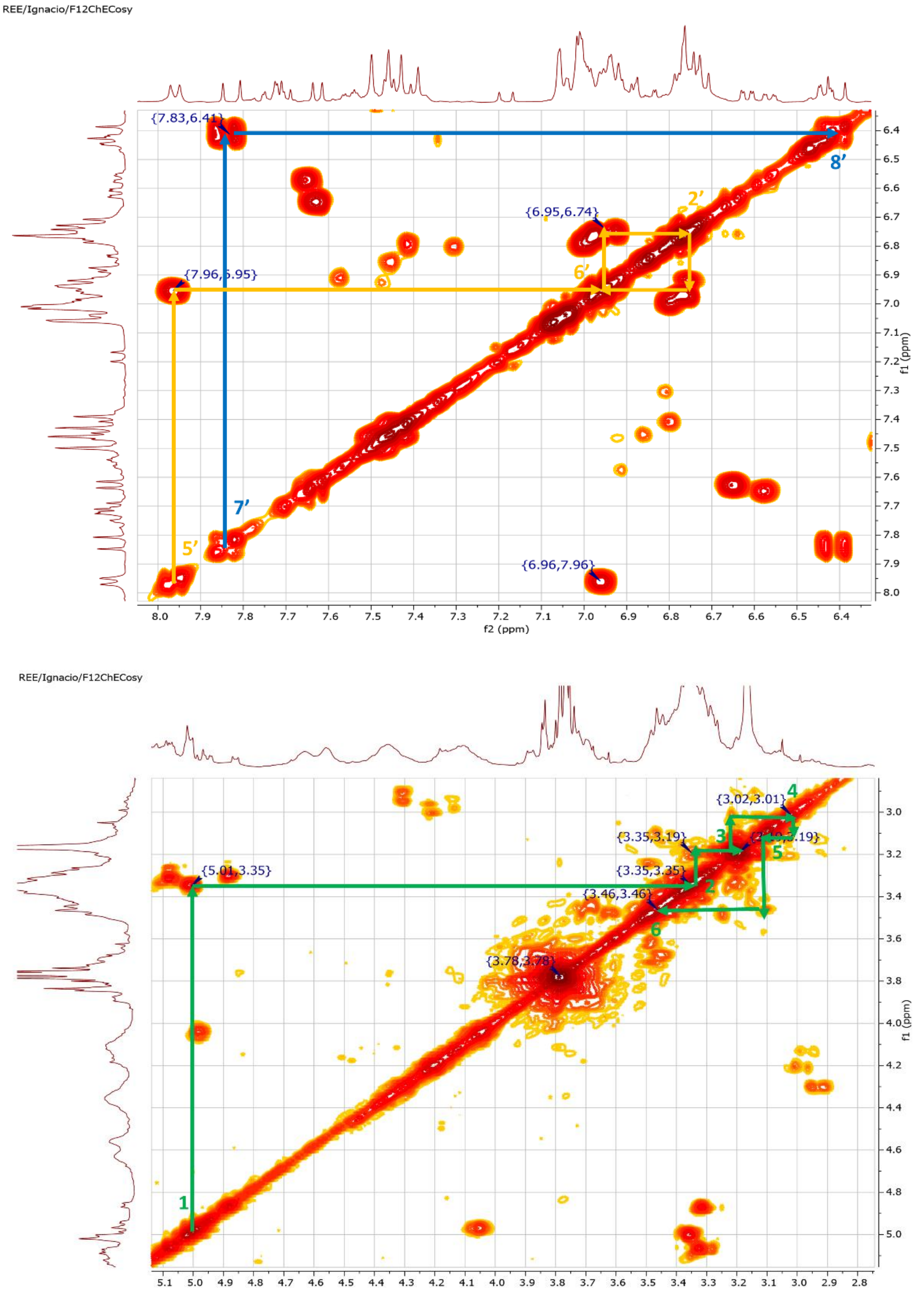

Figure S27: COSY NMR spectrum F12-ChE measured in DMSO-d_6 at 400 MHz. Blue line represents the propenoic acid moiety, yellow line is the ABX system of the 1,3,4-trisubstituted phenyl system of the ferulic acid unit, and the green line traces the coupling protons for the glucose unit.

Table S6: ^{1}H NMR data (δ_H in ppm, mult, *J* in Hz) of F12-ChE in comparison to that of 4'→1-*O*-feruloyl-β-D-glucose and 1-*O*- feruloyl-β-D- glucose as reported in the literature.

Atom no.	F12-ChE (DMSO-d_6) 400 MHz	4'→1-*O*- feruloyl-β-D- glucose [15] (CH_3CN:C_6D_6) 250 MHz	1-*O*-feruloyl-β-D-glucose [13] (MeOD) 400 MHz
1	5.01 d, *J* = 7.1	4.98 d, *J* = 7.2	5.56 d, *J* =7.8
2	3.41 – 3.29 m	3.20 – 3.60 m	3.40 – 3.50 m
3	3.24 – 3.10 m	3.20 – 3.60 m	3.40 – 3.50 m
4	3.09 – 2.93 m	3.20 – 3.60 m	3.40 – 3.50 m
5	3.24 – 3.10 m	3.20 – 3.60 m	3.4-3.5
6	3.53 – 3.40 m	3.64 dd, *J* = 12.0,5.0<br>3.84 dd, *J* = 12.1, 1.9	3.86 dd, *J* =1.9, 12.2
2'	6.73 d, *J* = 3.1	6.80 d, *J* = 2.3	7.20 d, *J* = 1.9
5'	7.96 d, *J* = 8.3	7.52 d, *J* = 8.6	6.81 d, *J* = 8.3
6'	6.95 dd, J = 3.1, 7.9	6.57 dd, *J* = 2.3, 8.6	7.09 dd, *J* = 1.9, 8.3
7'	7.83 d, *J* = 16.3	8.04 d, *J* = 16.0	7.61 d, *J* = 15.9
8'	6.41 d, *J* = 16.3	6.43 d, *J* = 16.0	6.39 d, *J* = 15.9
3'-OCH_3	3.78 s	3.76 s	3.89 s

References:

1. Horman, I.; Badoud, R.; Ammann, W. Food-related applications of one- and two-dimensional high-resolution proton-NMR: structure and conformation of cynarin. *Journal of Agricultural and Food Chemistry* **1984**, *32*, 538-540, doi:10.1021/jf00123a030.
2. Tolonen, A.; Joutsamo, T.; Mattlla, S.; Kamarainen, T.; Jalonen, J. Identification of isomeric dicaffeoylquinic acids from Eleutherococcus senticosus using HPLC-ESI/TOF/MS and 1H-NMR methods. *Phytochem Anal* **2002**, *13*, 316-328, doi:10.1002/pca.663.
3. Alwahsh, M.; Khairuddean, M.; Chong, W. Chemical constituents and antioxidant activity of *Teucrium barbeyanum* Aschers. *Rec Nat Prod* **2015**, *9*, 159-163.
4. Victor, M.; David, J.; Sakukuma, M.; França, E.; Nunes, A. A simple and efficient process for the extraction of naringin from grapefruit peel waste. *Green Processing and Synthesis* **2017**, *7*, doi:10.1515/gps-2017-0112.
5. Olennikov, D.N.; Kashchenko, N.I. New acylated apigenin glycosides from edge flowers of *Matricaria chamomilla*. *Chem Nat Compd* **2016**, *52*, 996-999, doi:10.1007/s10600-016-1845-7.
6. Wan, C.; Li, S.; Liu, L.; Chen, C.; Fan, S. Caffeoylquinic Acids from the Aerial Parts of *Chrysanthemum coronarium* L. *Plants (Basel)* **2017**, *6*, 10, doi:10.3390/plants6010010.
7. Horman, I.; Badoud, R.; Ammann, W. Food-related applications of one- and two-dimensional high-resolution proton-NMR: structure and conformation of cynarin. *J Agric Food Chem* **1984**, *32*, 538-540, doi:10.1021/jf00123a030.

8. de Falco, B.; Incerti, G.; Pepe, R.; Amato, M.; Lanzotti, V. Metabolomic fingerprinting of Romaneschi globe artichokes by NMR spectroscopy and multivariate data analysis: Metabolomics of artichokes by NMR and chemometrics. *Phytochem Anal* **2016**, *27*, 304-314, doi:10.1002/pca.2632.
9. Carlotto, J.; da Silva, L.M.; Dartora, N.; Maria-Ferreira, D.; Sabry, D.d.A.; Filho, A.P.S.; de Paula Werner, M.F.; Sassaki, G.L.; Gorin, P.A.J.; Iacomini, M.; et al. Identification of a dicaffeoylquinic acid isomer from *Arctium lappa* with a potent anti-ulcer activity. *Talanta* **2015**, *135*, 50-57, doi:https://doi.org/10.1016/j.talanta.2014.11.068.
10. Wan, C.; Li, S.; Liu, L.; Chen, C.; Fan, S. Caffeoylquinic acids from the aerial parts of *Chrysanthemum coronarium* L. *Plants (Basel)* **2017**, *6*, 10, doi:10.3390/plants6010010.
11. Tolonen, A.; Joutsamo, T.; Mattlla, S.; Kamarainen, T.; Jalonen, J. Identification of isomeric dicaffeoylquinic acids from *Eleutherococcus senticosus* using HPLC-ESI/TOF/MS and 1H-NMR methods. *Phytochem Anal* **2002**, *13*, 316-328, doi:10.1002/pca.663.
12. Pearson, W.A.; Spessard, G.O. α[alpha]- and β[bet]a-D-glucose pentaacetate. An experiment in structure assignment using NMR. *Journal of Chemical Education* **1975**, *52*, 814, doi:10.1021/ed052p814.
13. Takaya, Y.; Kondo, Y.; Furukawa, T.; Niwa, M. Antioxidant constituents of radish sprout (Kaiware-daikon), *Raphanus sativus* L. *J Agric Food Chem* **2003**, *51*, 8061-8066, doi:10.1021/jf0346206.
14. Galland, S.; Mora, N.; Abert-Vian, M.; Rakotomanomana, N.; Dangles, O. Chemical synthesis of hydroxycinnamic acid glucosides and evaluation of their ability to stabilize natural colors via anthocyanin copigmentation. *J Agric Food Chem* **2007**, *55*, 7573-7579, doi:10.1021/jf071205v.
15. Kurkin, V.A.; Lamrini, M.; Klochkov, S.G. Lavandoside from *Lavandula spica* flowers. *Chem Nat Compd* **2008**, *44*, 169-170, doi:10.1007/s10600-008-9005-3.
